# Increasing Dietary Medium-Chain Fatty Acid Ratio Mitigates High-fat Diet-Induced Non-Alcoholic Steatohepatitis by Regulating Autophagy

**DOI:** 10.1038/s41598-017-14376-y

**Published:** 2017-10-25

**Authors:** Mu-En Wang, Brijesh K. Singh, Meng-Chieh Hsu, Chien Huang, Paul M. Yen, Leang-Shin Wu, De-Shien Jong, Chih-Hsien Chiu

**Affiliations:** 1 Laboratory of Animal Physiology, Department of Animal Science and Technology, National Taiwan University, Taipei, 10617 Taiwan; 2 Laboratory of Hormonal Regulation, Cardiovascular and Metabolic Disorders Program, Duke-National University of Singapore Medical School, Singapore, 16987 Singapore

## Abstract

Previous studies have demonstrated that saturated fatty acids (SFAs) are more lipotoxic than unsaturated fatty acids (UFAs) in inhibiting hepatic autophagy and promoting non-alcoholic steatohepatitis (NASH). However, there have been few studies have investigated the effects of carbon chain length on SFA-induced autophagy impairment and lipotoxicity. To investigate whether SFAs with shorter carbon chain lengths have differential effects on hepatic autophagy and NASH development, we partially replaced lard with coconut oil to elevate the ratio of medium-chain fatty acids (MCFAs) to long-chain fatty acids (LCFAs) in a mouse high-fat diet (HFD) and fed mice for 16 weeks. In addition, we treated HepG2 cells with different combinations of fatty acids to study the mechanisms of MCFAs-mediated hepatic protections. Our results showed that increasing dietary MCFA/LCFA ratio mitigated HFD-induced Type 2 diabetes and NASH in mice. Importantly, we demonstrated that increased MCFA ratio exerted its protective effects by restoring Rubicon-suppressed autophagy. Our study suggests that the relative amount of LCFAs and MCFAs in the diet, in addition to the amount of SFAs, can significantly contribute to autophagy impairment and hepatic lipotoxicity. Collectively, we propose that increasing dietary MCFAs could be an alternative therapeutic and prevention strategy for Type 2 diabetes and NASH.

## Introduction

The transition from simple steatosis to non-alcoholic steatohepatitis (NASH) significantly increases the risk of advanced cirrhosis, liver failure, and hepatocellular carcinoma. Thus, NASH is currently considered to be the determining stage in the progression of non-alcoholic fatty liver diseases (NAFLD), and its prevention is becoming an important clinical issue. Although previous clinical and animal studies have suggested obesity, insulin resistance, and Type 2 diabetes are important risk factors for NASH^1–5^, the pathogenic mechanisms of NASH are still unclear.

Recent studies have found that hepatic autophagy plays an important role in human and mouse NAFLD progression by regulating lipid metabolism, endoplasmic reticulum (ER) stress, and cell apoptosis in hepatocytes^6–9^. Autophagy is an important cellular process regulating metabolic homeostasis and stress responses. Under different nutrient conditions, the AMPK/mTOR/ULK1 pathway is tightly controlled to regulate early-stage induction of autophagy^10^. During early-stage autophagy induction, the newly synthesized double-membrane autophagosomes engulf damaged and redundant intracellular components such as organelles and proteins. Mature autophagosomes then will fuse with lysosomes to form autolysosomes for cargo degradation^11^. Although impaired autophagy was found to be highly correlated with NAFLD progression, the major triggers and molecular mechanisms that lead to decreased autophagy remain unclear. According to previous studies, both early-stage and late-stage autophagy suppression occur during NAFLD development. Previously, fatty acids were shown to inhibit AMPK- or mTOR-regulated autophagy in rat and human hepatocytes^12,13^. Additionally, recent studies demonstrated late-stage inhibition of autophagy via impaired lysosomal acidification^14,15^ or reduced autophagosome-lysosome fusion^16^.

The roles of saturated fatty acids (SFAs) in promoting insulin resistance, autophagy impairment, and NAFLD have been well characterized^6,16–20^. Additionally, many recent studies have shown the protective effects of unsaturated fatty acids (UFAs) on NAFLD^21–23^. However, little is known currently about the role of fatty acid carbon chain length on NASH development. Medium-chain fatty acids (MCFAs) are fatty acids containing 6 to 12 carbons that are commonly found in coconut oil and animal milk^24^. Previous reports indicated that MCFAs improved Type 2 diabetes and NAFLD^25,26^, but the molecular mechanisms underlying these effects were not well characterized. Accordingly, we investigated the potential protective effects of MCFAs on NASH using mouse and cell culture models. In particular, we examined whether increasing the MCFA/long chain fatty acid (LCFA) ratio in HFD protected against Type 2 diabetes and NASH by regulating autophagy.

## Results

### Increasing dietary MCFA ratio delayed the development of Type 2 diabetes in mice

**Figure 1:**
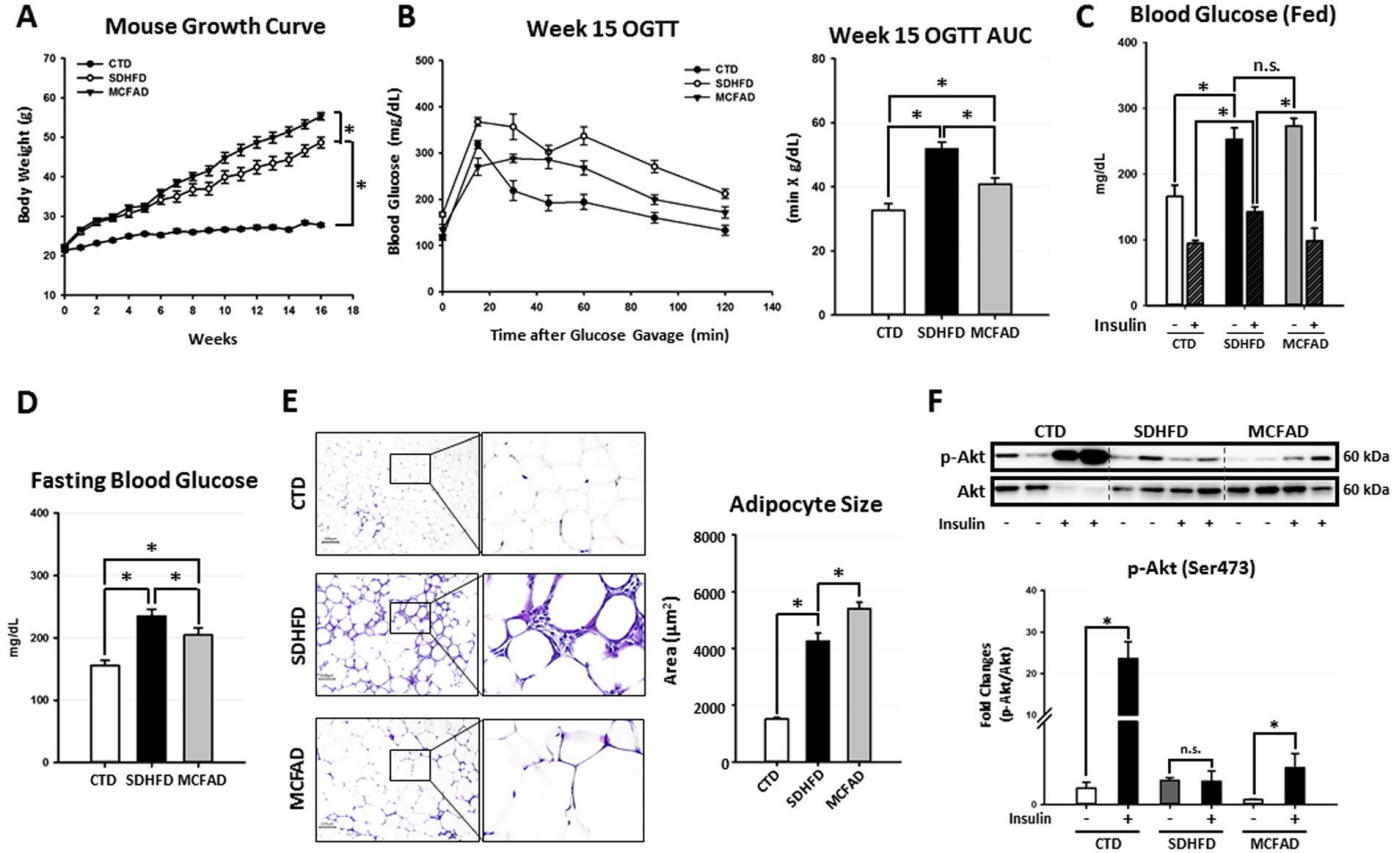
Increasing dietary MCFA ratio delayed Type 2 diabetes development in HFD-fed mice. Eight-week-old male C57BL/6 mice were fed with either the control low-fat diet (CTD), standard high-fat diet (SDHFD), or MCFAs-rich high-fat diet (MCFAD) for 16 weeks. During the experiment, mouse body weight was checked every week (**A**). Development of systemic insulin resistance and Type 2 diabetes was analyzed by conducting OGTT test at the 15^th^ week (**B**) and measuring fed blood glucose levels before and after I.P. insulin injection (0.5 IU/kg body weight, 30 min) (**C**), as well as starving blood glucose at the 16^th^ week (**D**). Adipocyte cell size in H&E-stained sections was quantified automatically using Adiposoft software (**E**). For adipose insulin resistance, the phospho-Akt (Ser473) levels of adipose tissues from mice injected with or without I.P. insulin (0.5 IU/kg body weight, 30 min) were analyzed. Representative immunoblots and densitometric quantification of phospho-Akt (Ser473) are presented (**F**). Values are mean ± SEM. (n = 6). *Indicates statistical significance, *P* < 0.05. n.s.: no significant difference. Full-length blots are presented in Supplementary Figure S1.

To confirm whether increasing dietary MCFA/LCFA ratio could protect against HFD-induced Type 2 diabetes and NASH, we fed male C57BL/6 mice with either low-fat control diet (CTD), standard high-fat diet (SDHFD), or MCFA-rich high-fat diet (MCFAD) for 16 weeks. As shown in Fig. 1A, both SDHFD and MCFAD significantly increased mouse body weight after the first week of treatment. After the 6^th^ week, the MCFAD group had higher weight gain than the SDHFD group. Surprisingly, despite the increased weight gain, 15-week MCFAD-fed mice showed significantly improved oral glucose tolerance than SDHFD-fed mice (Fig. 1B). To further test whether MCFAs also improved insulin sensitivity, we injected mice with a single dose of insulin (0.5 IU/kg body weight) intraperitoneally for 30 minutes at the 16^th^ week and compared the capability of insulin to reduce blood glucose levels in fed mice between different treating groups. Glucose concentrations were increased to similar levels in both SDHFD- and MCFAD-fed mice, whereas insulin reduced blood glucose levels in MCFAD-fed mice more than those fed with SDHFD (Fig. 1C). Although glucose was elevated in mice that were fed with either diet, mice fed with MCFAD had significantly lower fasting glucose levels than those fed with SDHFD (Fig. 1D).

Since MCFAD-fed mice showed less systemic insulin resistance than SDHFD-fed mice, it is possible that their increased weight gain could be due to attenuated adipose insulin resistance, which is known to cause elevated lipolysis and decreased lipid storage in adipose tissues^27^. We found that MCFAD-fed mice had significantly larger adipose cell size compared to SDHFD-fed mice (Fig. 1E). Besides, H&E-stained sections of adipose tissue showed smaller adipocytes that were surrounded by macrophages in mice fed SDHFD compared to uniformly large adipocytes with no obvious macrophage infiltration in mice fed MCFAD (Fig. 1E). More importantly, MCFAD partially restored the capability of insulin to induce Akt phosphorylation (Ser473) in adipose tissues of HFD-fed mice (Fig. 1F). These results indicated that MCFAD-fed mice had less adipose insulin resistance and inflammation than SDHFD-fed mice. Taken together, our data strongly suggested that increasing the dietary MCFA/LCFA ratio decreased the severity of HFD-induced Type 2 diabetes in mice.

### Increasing dietary MCFA ratio mitigated hepatic autophagy impairment and NASH development in HFD-fed mice

**Figure 2:**
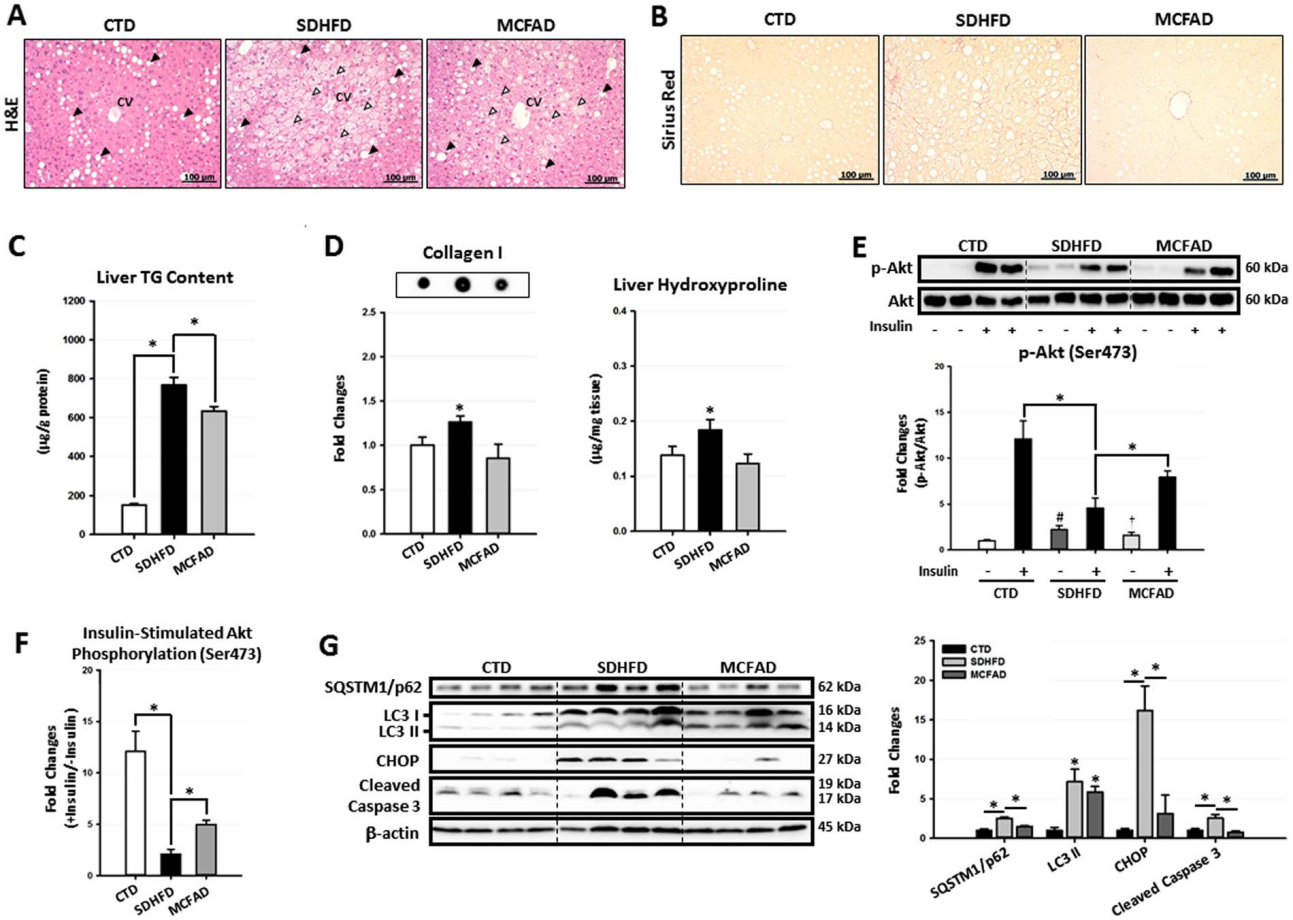
Increasing dietary MCFA ratio attenuated NASH development in mice. To investigate the protective effects of MCFAs on NASH development, paraffin-embedded mouse liver sections were stained with H&E and Sirius Red to evaluate hepatic steatosis (**A**) and fibrosis (**B**) respectively. Hepatic TG content (**C**), collagen I protein and hydroxyproline (**D**) levels were also quantified using a commercial assay kit and dot blotting assay. Hepatic insulin resistance, autophagic flux, ER stress, and apoptosis were analyzed using Western blotting. For insulin resistance, the hepatic phospho-Akt (Ser473) level in mice injected with or without I.P. insulin (0.5 IU/kg body weight, 30 min) were analyzed. Representative immunoblots and densitometric quantification of phospho-Akt (Ser473) (**E**), and the calculated insulin-stimulated Akt phosphorylation fold change results (**F**) are presented. Representative SQSTM1/p62, LC3, CHOP, and cleaved caspase 3 immunoblots and densitometric quantifications (**G**) indicate that increasing MCFA ratio attenuated HFD-induced hepatic autophagy impairment, ER stress, and cell apoptosis. For densitometric analyses of Western blotting data, β-actin and Akt (for Akt phosphorylation analysis) were used as the loading controls. Values are mean ± SEM (n = 6). *, ^#^(vs. CTD fed mice without insulin injection), and ^†^(vs. SDHFD fed mice without insulin injection) indicate statistical significance, *P* < 0.05. Full-length blots are presented in Supplementary Figure S2.

Because the progression of NASH is commonly associated with the development of insulin resistance and Type 2 diabetes, we next investigated whether increasing the dietary MCFA/LCFA ratio protected against HFD-induced NASH. In Fig. 2A, H&E-stained liver sections showed that mice from all three groups exhibited zone 1 macrovesicular steatosis, which was most likely due to post-starvation lipid droplet formation^28^. However, when mice were fed with HFD, zone 3 microvesicular steatosis was profoundly increased. This zone was recently found to be correlated with more severe liver injuries and advanced NAFLD stages^29^. Of note, the zone 3 microvesicular steatosis was significantly decreased in MCFAD-fed mice when compared to SDHFD-fed mice. Additionally, MCFAD-fed mice showed lower liver TG accumulation compared to SDHFD group (Fig. 2C). More importantly, we found that increasing dietary MCFA ratio significantly decreased HFD-induced hepatic fibrosis, an important hallmark of NASH. Sirius Red-stained liver sections showed that pericellular fibrosis was significantly induced in SDHFD-fed mice, whereas little hepatic fibrosis was found in MCFAD-fed mice (Fig. 2B). Consistently, SDHFD- but not MCFAD-fed mice had significantly increased hepatic collagen I and hydroxyproline accumulation (Fig. 2D). Taken together, our data demonstrated that increasing dietary MCFA/LCFA ratio significantly attenuated HFD-induced NASH in mice.

There is a growing body of evidence showing that hepatic insulin resistance and autophagy impairment are strongly associated with NASH development^6,7,16,30^. Accordingly, we next examined the protective effects of MCFAs on HFD-induced hepatic insulin resistance and autophagy impairment. We found that long-term SDHFD feeding significantly increased the basal phospho-Akt level but decreased insulin-stimulated Akt phosphorylation in mouse livers. Interestingly, we found that MCFAD-fed mice had decreased basal phospho-Akt level but enhanced insulin-stimulated Akt phosphorylation when compared to SDHFD group (Fig. 2E). In other words, increasing dietary MCFA ratio partially restored the capacity of insulin to stimulate hepatic Akt phosphorylation that was reduced by HFD feeding (Fig. 2F).

We next examined the effects of MCFAD on hepatic autophagy. As shown in Fig. 2G, the autophagosome marker protein LC3 II was significantly increased in both SDHFD- and MCFAD-fed mice livers. However, when compared to SDHFD, MCFAD induced less accumulation of hepatic SQSTM1/p62, a cargo protein delivering ubiquitinated substrates into autophagosome for degradation^31^. These data suggested that HFD decreased hepatic autophagy; however, increasing dietary MCFA/LCFA ratio mitigated HFD-induced autophagy impairment in the liver. Additionally, we found that the improvement in autophagy also led to attenuation of hepatic ER stress and apoptosis caused by long-term HFD feeding. As shown in Fig. 2G, the inductions of ER stress marker, CHOP, and the apoptosis marker, cleaved caspase 3, were less in the livers of MCFAD-fed mice compared to SDHFD-fed mice.

### Restoration of hepatic autophagy by increasing MCFA ratio attenuated fat accumulation, insulin resistance, and lipotoxicity caused by LCFAs

**Figure 3:**
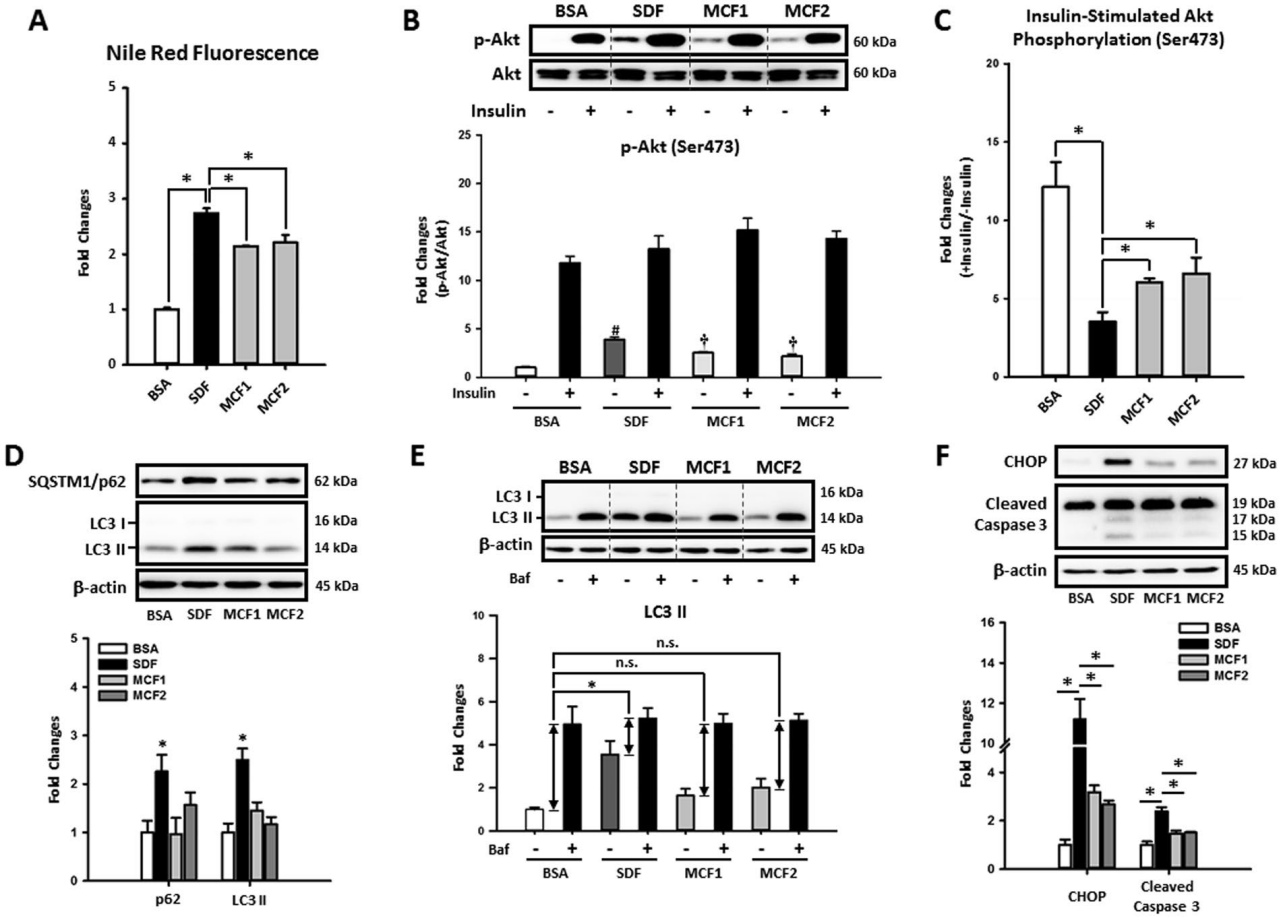
Increasing MCFA ratio attenuated LCFAs-induced fat accumulation, insulin resistance, autophagy impairment, ER stress, and apoptosis in HepG2 cells. To investigate whether MCFAs can directly exert their protective effects in hepatic cells, the fat accumulation, insulin resistance, autophagy impairment, ER stress, and apoptosis were evaluated in fat-loaded HepG2 cells. For intracellular lipid quantification, 48-hour fat-loaded cells were fixed then stained with Nile Red and Hoechst 33342 (**A**). For the insulin sensitivity test, cells were incubated with 100 nM insulin for an additional 10 min after a 48-hour fatty acid treatment. Representative immunoblots and densitometric quantification of phospho-Akt (Ser473) (**B**), and the calculated insulin-stimulated Akt phosphorylation fold change results (**C**) are presented. Representative SQSTM1/p62 and LC3 immunoblots and densitometric results show that increasing MCFA/LCFA ratio protected HepG2 cells against LCFA-induced autophagy impairment (**D**). Autophagic flux changes were also confirmed by analyzing Bafilomycin A1 (50 nM, 3 hours)-induced LC3 II protein accumulation in BSA or fatty acid-treated HepG2 cells (**E**). Western blotting results of CHOP and cleaved caspase 3 show that MCFAs also attenuated LCFAs-induced ER stress and apoptosis in HepG2 cells (**F**). For densitometric analyses of Western blotting data, β-actin or total Akt (for Akt phosphorylation analysis) were used as the loading control. Values are mean ± SEM (n = 3). *, ^#^(vs. BSA treated cells without insulin stimulation), and ^†^(vs. SDF-treated cells without insulin stimulation) indicate statistical significance, *P* < 0.05. n.s.: no significant difference. SDF: standard fat loading mixture (0.4 mM PA + 0.4 mM OA); MCF1: MCFA-containing fat mixture 1 (0.4 mM PA + 0.2 mM OA + 0.2 mM CA); MCF2: MCFA-containing fat mixture 2 (0.4 mM PA + 0.2 mM OA + 0.2 mM LA); Baf: Bafilomycin A1. Full-length blots are presented in Supplementary Figure S3.

To investigate whether increasing MCFA/LCFA ratio protected hepatic cells against LCFA-induced steatosis and lipotoxicity in a cell-autonomous manner, we conducted a series of experiments using the HepG2 cell line, which has some metabolic characteristics that resemble human liver tissue^32^ and was used in our previous NAFLD studies^33,34^. We first checked the protective effects of increasing MCFA/LCFA ratio on HepG2 cells by treating cells with different fatty acid mixtures for 48 hours. Briefly, we treated cells with the mixture of 0.4 mM PA and 0.4 mM OA (designated as SDF, the standard fat loading mixture) to simulate the lipotoxicity caused by LCFAs. To evaluate the protective effects of increasing MCFA/LCFA ratio on hepatic cells, we replaced half of the OA in SDF mixture with capric acid (C10, CA) or lauric acid (C12, LA), which were designated as MCFA mixture 1 (MCF1) and MCFA mixture 2 (MCF2), respectively. Similar to our *in vivo* findings, increasing the MCFA ratio significantly decreased the amount of fat accumulation in HepG2 cells during fat loading (Fig. 3A). In addition, Western blotting results showed that insulin resistance also was attenuated in MCF1- and MCF2-treated cells. Results in Fig. 3B showed that SDF treatment did not lower insulin-stimulated Akt phosphorylation (Ser473); however, it increased the basal phospho-Akt level that was found to play an important role in HFD-desensitized hepatic insulin signaling^35^. On the other hand, both MCF1- and MCF2-treated cells showed decreased basal phospho-Akt levels when compared to SDF-treated cells while maintaining a similar level of insulin-stimulated Akt phosphorylation. Thus, these data indicated that increasing the MCFA ratio restored the capacity of insulin to stimulate Akt phosphorylation in HepG2 cells (Fig. 3C). Similar results were also found in GSK-3β phosphorylation (Ser9) as shown in Figure S9.

Our data also showed that increasing MCFA ratio rescued autophagy impairment, ER stress, and apoptosis in hepatic cells caused by SDF. As shown in Fig. 3D, the accumulation of both LC3 II and SQSTM1/p62 proteins in fat-loaded HepG2 cells was significantly reduced when the MCFA ratio was increased, indicating a restoration of autophagic flux. The autophagic flux changes also were confirmed by measuring LC3 II accumulation induced by the late-stage autophagy blocker Bafilomycin A1. Although SDF treatment increased the basal LC3 II protein level in HepG2 cells, the Bafilomycin A1-induced LC3 II accumulation in SDF-loaded cells was less than BSA-treated cells, indicating inhibited autophagic flux (Fig. 3E). Consistent with the previous SQSTM1/p62 results of cell culture and *in vivo* experiments, increasing MCFA/LCFA ratio fully restored the autophagic flux in HepG2 cells (Fig. 3E). Similar to our *in vivo* data, ER stress and apoptosis caused by SDF treatment were also decreased after restoration of autophagy by MCFA (Fig. 3F).

**Figure 4:**
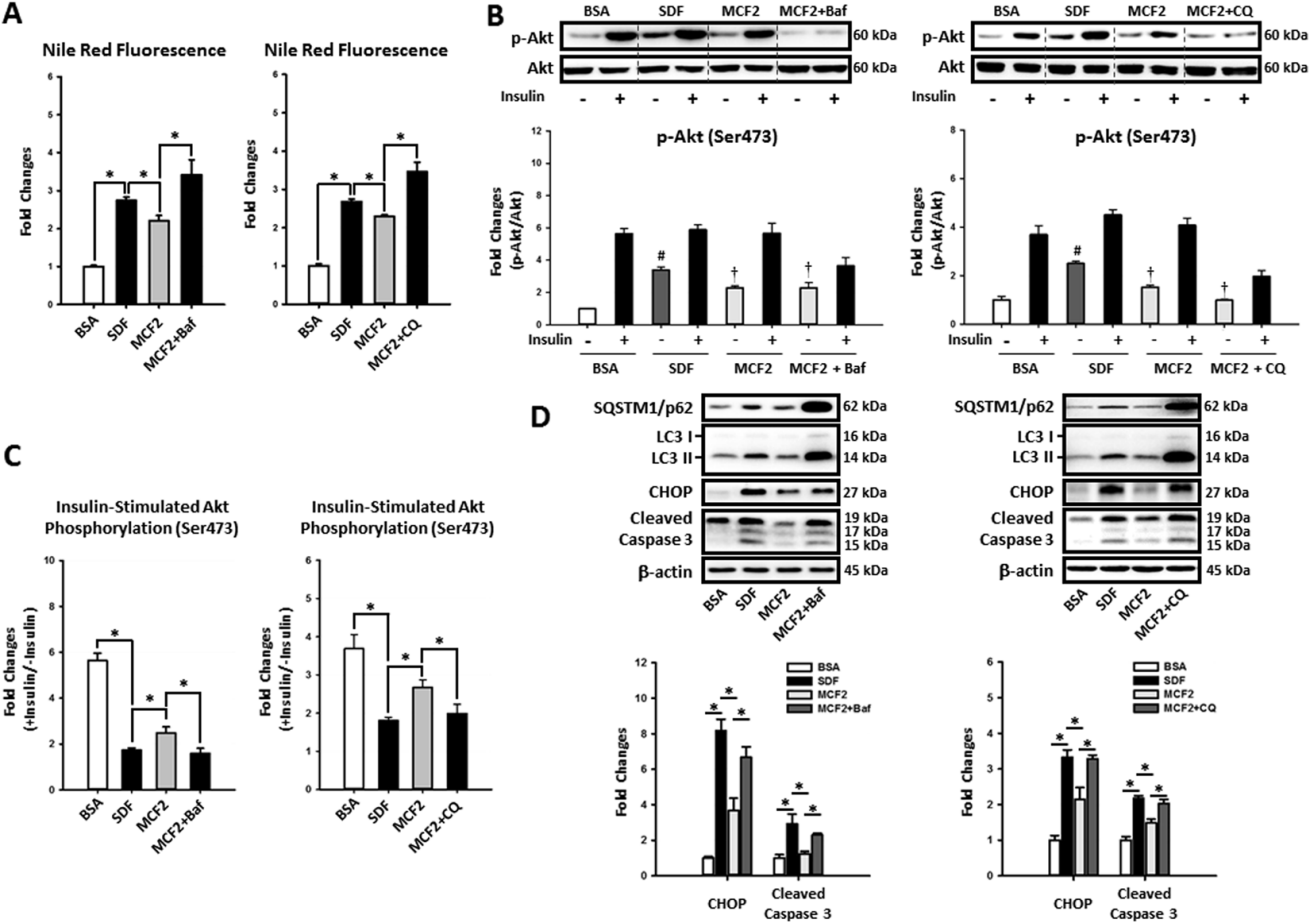
Increasing MCFA ratio protected HepG2 cells against LCFAs-induced fat accumulation, insulin resistance, and lipotoxicity by rescuing autophagy. To investigate the role of autophagy in MCFA-mediated protections, we blocked autophagy in MCF2-loaded HepG2 cells using either Bafilomycin A1 (5 nM) or chloroquine (10 μM). After treatment, fat accumulation (**A**), insulin resistance (**B** and **C**), ER stress, and apoptosis (**D**) were analyzed. Values are mean ± SEM (n = 3). For densitometric analyses of Western blotting data, β-actin or total Akt (for Akt phosphorylation analyses only) were used as the loading controls. *, ^#^(vs. BSA treated cells without insulin stimulation), and ^†^(vs. SDF treated cells without insulin stimulation) indicate statistical significance, *P* < 0.05. n.s.: no significant difference. SDF: standard fat loading mixture (0.4 mM PA + 0.4 mM OA); MCF2: MCFA-containing fat mixture 2 (0.4 mM PA + 0.2 mM OA + 0.2 mM LA); Baf: Bafilomycin A1. CQ: chloroquine. Full-length blots are presented in Supplementary Figure S4.

Since many previous studies have proposed that autophagy plays an important role in NASH development^6,7,16^, we hypothesized that increasing the MCFA/LCFA ratio may protect hepatic cells against LCFA-induced steatosis, insulin resistance, and lipotoxicity by regulating autophagy. To confirm our hypothesis, we blocked the autophagic flux in MCF2-treated HepG2 cells using Bafilomycin A1 and chloroquine. Indeed, when autophagic flux was blocked, increasing the MCFA ratio was no longer able to protect HepG2 cells against fat accumulation (Fig. 4A), insulin resistance (Fig. 4B,C), ER stress, and apoptosis caused by LCFAs (Fig. 4D). Collectively, these data suggest that autophagy was needed for MCFA-mediated protection in hepatic cells.

### MCFAs protected hepatic cells against LCFAs-induced autophagy impairment by regulating Rubicon instead of the AMPK/mTOR/ULK1 pathway

**Figure 5:**
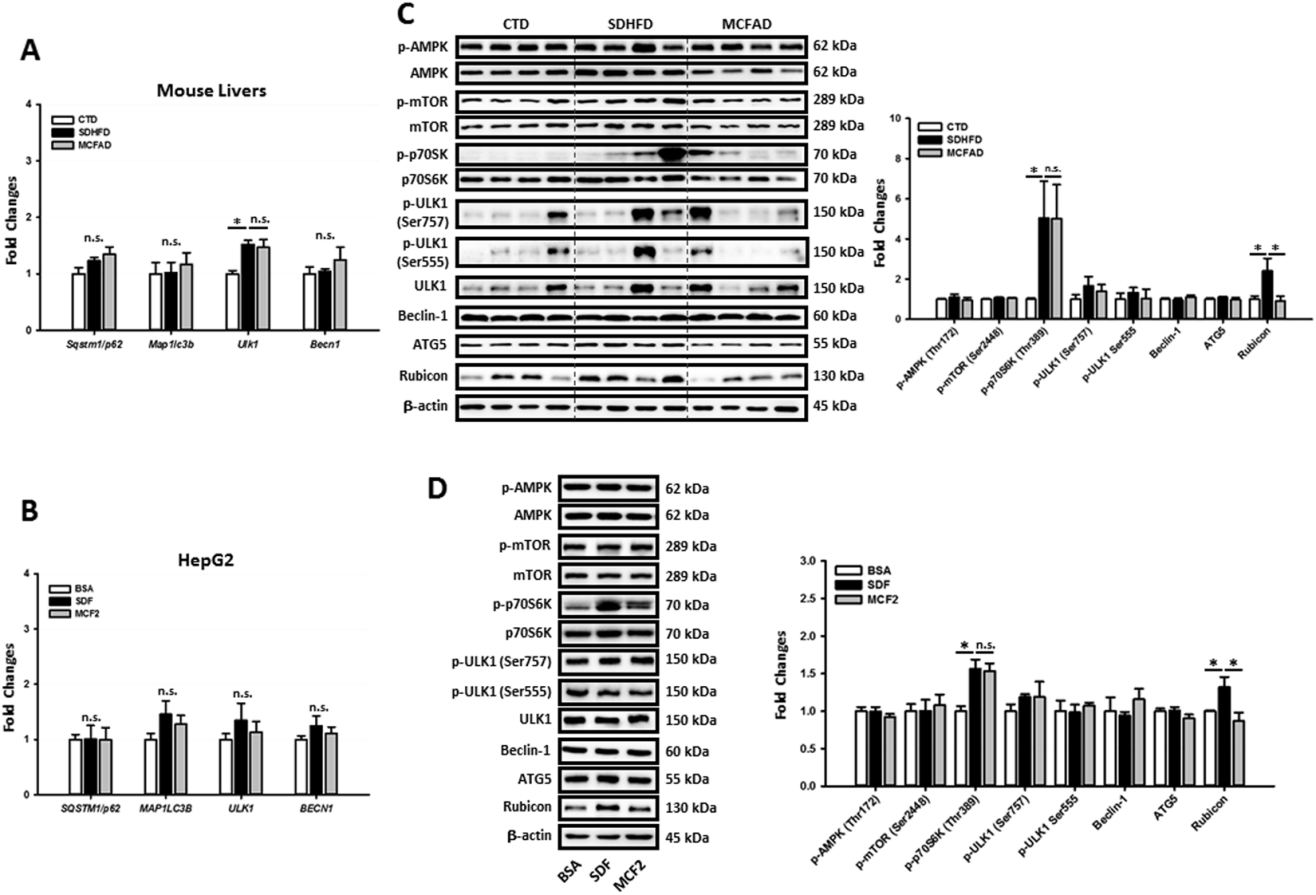
Increasing MCFA ratio restored hepatic autophagy by downregulating LCFA-induced Rubicon independently of regulating early-stage autophagy signaling pathways. To clarify the molecular mechanisms of MCFAs-protected autophagy, we analyzed the regulations on early-stage and late-stage autophagy signaling pathways in both mouse livers and HepG2 cells. The *Sqstm1/p62, Map1lc3b, Ulk1, Becn1, SQSTM1/p62, MAP1LC3B, ULK1*, and* BECN1* mRNA levels in mouse livers (**A**) and HepG2 cells (**B**) were measured using qPCR. The expression of phospho-AMPK (Thr172) phospho-mTOR (Ser2448), phospho-p70S6K (Thr389), phospho-ULK1 (Ser757), phospho-ULK1 (Ser555), Beclin1, ATG5, and Rubicon in HFD-fed mouse livers (**C**) and fat-loaded HepG2 cells (**D**) are analyzed using Western blotting. For qPCR, *Actb* and *ACTB* mRNA levels were used as the internal controls. For densitometric analyses of Western blotting data, β-actin and total target protein (for phospho-proteins) were used as the loading controls. Values are mean ± SEM (n = 6 and 3 for *in vivo* and *in vitro* studies respectively). ^*^ *P* < 0.05. n.s.: no significant difference; SDF: standard fat loading mixture (0.4 mM PA + 0.4 mM OA); MCF2: MCFA-containing fat mixture 2 (0.4 mM PA + 0.2 mM OA + 0.2 mM LA). Full-length blots are presented in Supplementary Figure S5.

**Figure 6:**
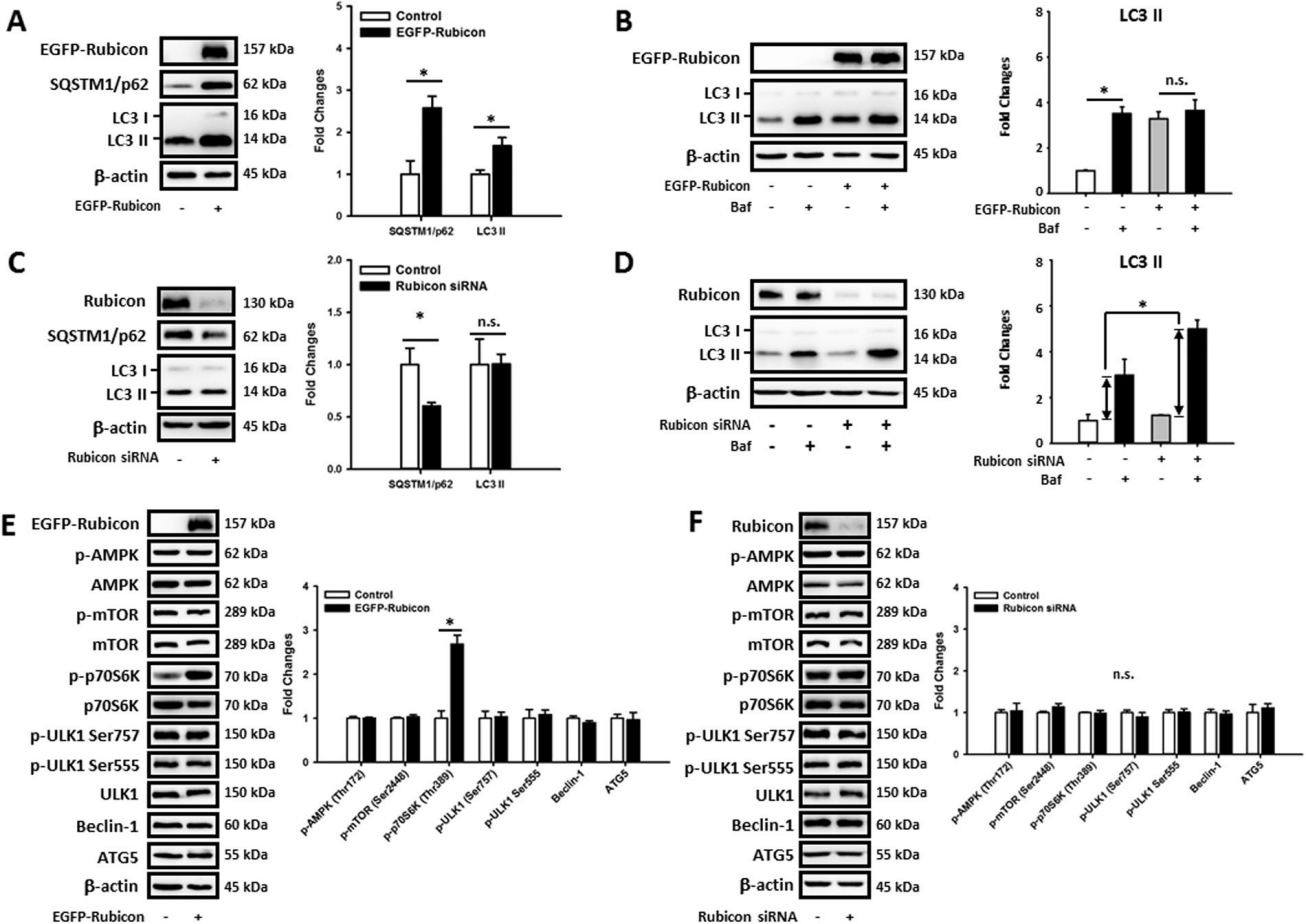
Rubicon inhibits late-stage autophagy activity in HepG2 cells independently of regulating the AMPK/mTOR/ULK1 signaling pathway. To investigate the regulatory function of Rubicon on hepatic autophagy, we overexpressed or knocked down Rubicon in HepG2 cells by transfecting the EGFP-Rubicon plasmid or Rubicon targeting siRNA respectively. The Autophagic flux changes in Rubicon-overexpressed (**A** and **B**) or knocked-down cells (**C** and **D**) were analyzed using Western blotting. Representative immunoblots and densitometric results of phospho-AMPK (Thr172) phospho-mTOR (Ser2448), phospho-p70S6K (Thr389), phospho-ULK1 (Ser757), phospho-ULK1 (Ser555), Beclin1, ATG5 in Rubicon overexpressed (**E**) or knocked-down (**F**) HepG2 cells are shown. For densitometric analyses of Western blotting data, β-actin and total target protein (for phospho-proteins) were used as the loading controls. Values are mean ± SEM (n = 3). ^*^ *P* < 0.05. n.s.: no significant difference. Full-length blots are presented in Supplementary Figure S6.

The reverse of concomitant increases in SQSTM1/p62 and LC3 II protein levels of MCF1- and MCF2-treated HepG2 cells suggested that MCFAs might rescue autophagy at a late rather than early stage. Our qPCR data in Fig. 5A showed that long-term SDHFD treatment had no effect on the mRNA expression of *Map1lc3b*, *Sqstm1/p62*, and *Becn1*, but increased *Ulk1* in mouse livers. However, SDHFD- and MCFAD-fed mice had similar expressions of these genes. Additionally, *MAP1LC3B*, *SQSTM1/p62*, *ULK1*, and *BECN1* expressions were not significantly changed in either SDF- or MCF2-treated HepG2 cells (Fig. 5B). Thus, these data suggested that restoration of hepatic autophagy by increasing MCFAs did not involve the expression of these early-stage autophagy genes. We next checked the involvement of AMPK/mTOR/ULK1 pathway in MCFAs-regulated autophagy. As shown in Fig. 5C, long-term feeding of both SDHFD and MCFAD did not alter the phosphorylation of AMPK (Thr172), mTOR (Ser2448), and ULK1 (both Ser757 and Ser555) in mouse livers. Additionally, SDHFD and MCFAD did not change the protein levels of Beclin-1 and ATG5. Of note, although SDHFD increased the phosphorylation of a mTOR downstream target p70S6K (Thr389), MCFAD did not reverse it. Similar results also were found in SDF- and MCF2-treated HepG2 cells (Fig. 6D). Collectively, these data indicated that increasing MCFA/LCFA ratio protected hepatic cells from autophagy impairment without necessarily involving regulation of the early-stage autophagy signaling pathways. Since it was recently reported that HFD-upregulated hepatic Rubicon, a negative regulator of late-stage autophagosome maturation^36^, accelerates NAFLD development^16^, we next examined the involvement of Rubicon in MCFAs-rescued autophagy. We observed that LCFAs increased hepatic Rubicon level and increasing MCFA/LCFA ratio reversed the Rubicon protein accumulation in both mouse livers and HepG2 cells (Fig. 5C,D). To confirm whether downregulation of Rubicon was needed for MCFAs-restored autophagy, we overexpressed or knocked down Rubicon in HepG2 cells. Overexpression of Rubicon simultaneously increased the SQSTM1/p62 and LC3 II protein levels in HepG2 cells (Fig. 6A). In addition, Bafilomycin A1 did not further increase LC3 II accumulation in Rubicon-overexpressed cells (Fig. 6B) suggesting autophagic flux was maximally inhibited. On the contrary, Rubicon knockdown decreased SQSTM1/p62 protein accumulation without altering LC3 II protein level (Fig. 6C) but increased Bafilomycin A1 induced-LC3 II accumulation in HepG2 cells (Fig. 6D). These data suggested that Rubicon inhibited late-stage rather than early-stage autophagy in hepatic cells. Additionally, Rubicon suppressed autophagy independently of AMPK/mTOR/ULK1-regulated autophagy. As shown in Fig. 6E,F, the protein levels of phospho-AMPK (Thr172), phospho-mTOR (Ser2448), phospho-ULK1 (Ser555), phospho-ULK1 (Ser757), Beclin1, and ATG5 were not changed by either Rubicon overexpression or knockdown. Importantly, although Rubicon overexpression increased phospho-p70S6K, Rubicon knockdown did not decrease the phospho-p70S6K level in HepG2 cells.

**Figure 7:**
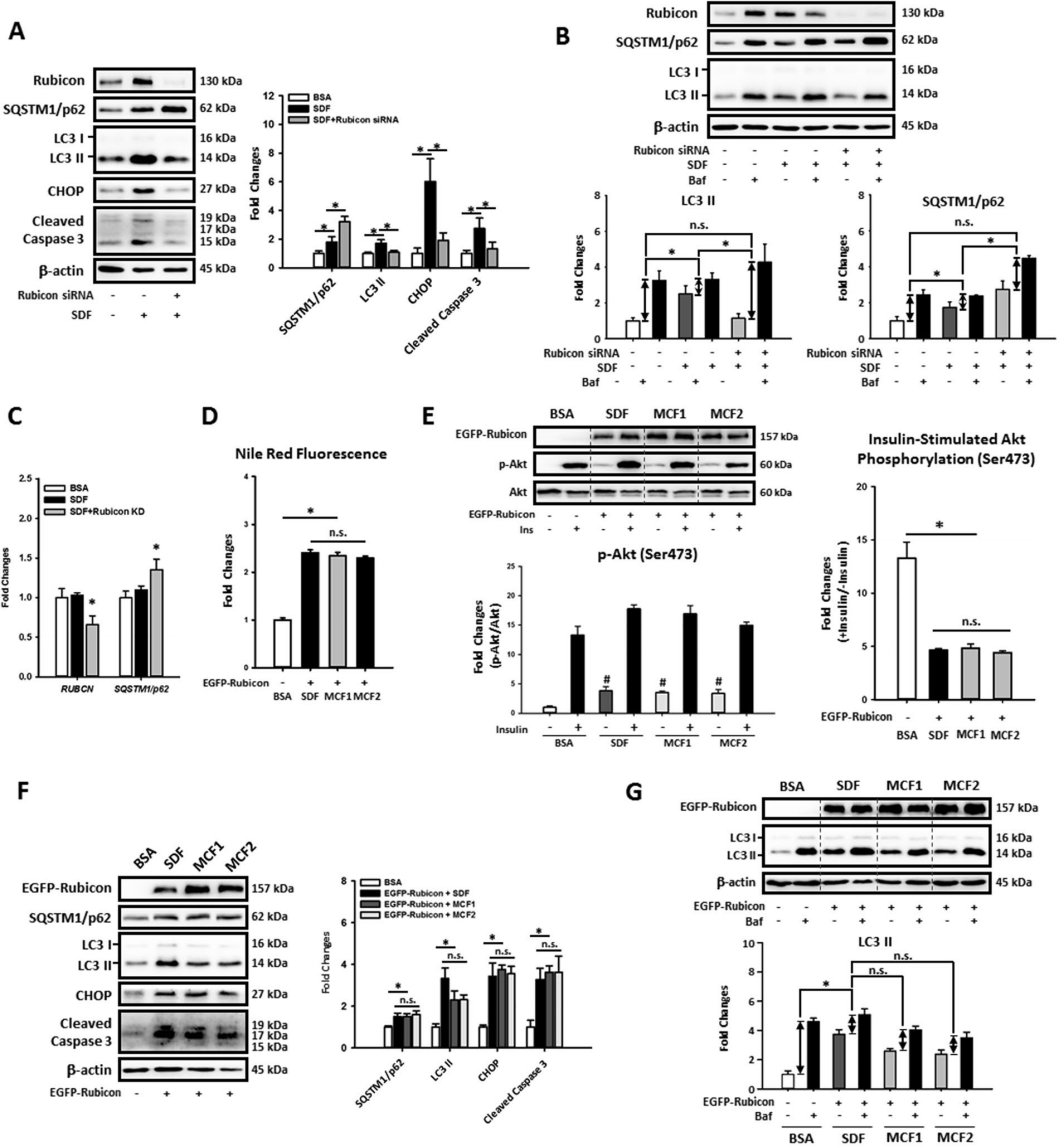
Increasing MCFA ratio protected HepG2 cells against LCFAs-induced fat accumulation, insulin resistance, autophagy impairment and lipotoxicity by downregulating Rubicon. To further confirm the involvement of Rubicon in MCFAs mediated protections, we knocked down or overexpressed Rubicon in fat-loaded HepG2 cells. The Western blotting results of CHOP and cleaved caspase 3 show that Rubicon knockdown potently attenuated LCFAs-induced lipotoxicity in HepG2 cells (**A**). Although Rubicon knockdown increased the SQSTM1/p62 protein level in fat-loaded cells, autophagic flux and qPCR assay results showed that Rubicon knockdown increased autophagic flux (**B**) and SQSTM1/p62 mRNA expression (**C**) simultaneously. Nile Red staining and Western blotting data also show that increasing MCFA ratio failed to rescued LCFAs-induced lipid accumulation (**D**), insulin resistance (**E**), autophagy impairment (**F,G**), and lipotoxicity (**F**) in Rubicon-overexpressed HepG2 cells, indicating that the downregulation of Rubicon is needed for MCFAs-mediated protections. For densitometric analyses of Western blotting data, β-actin was used as the loading control. Values are mean ± SEM (n = 3). ^*^ *P* < 0.05. n.s.: no significant difference. SDF: standard fat loading mixture (0.4 mM PA + 0.4 mM OA); MCF1: MCFA-containing fat mixture 1 (0.4 mM PA + 0.2 mM OA + 0.2 mM CA); MCF2: MCFA-containing fat mixture 2 (0.4 mM PA + 0.2 mM OA + 0.2 mM LA); Baf: Bafilomycin A1. Full-length blots are presented in Supplementary Figure S7.

We next investigated whether down-regulation of Rubicon and restoration of autophagy by MCFAs protected hepatic cells from lipotoxicity caused by LCFAs. As shown in Fig. 7A, Rubicon knockdown reversed LCFAs induction of LC3 II, CHOP, and cleaved caspase 3 protein levels in HepG2 cells. Of note, the SQSTM1/p62 protein level in fat-loaded cells was further increased by Rubicon knockdown; however, mRNA expression also was increased (Fig. 7C). Rubicon knockdown further increased Bafilomycin A1 induced LC3 II and p62 accumulations in fat-loaded HepG2 cells suggesting an increased autophagic flux after Rubicon knockdown (Fig. 7B). Importantly, we found that when Rubicon was overexpressed in HepG2 cells, the fat accumulation, insulin resistance, autophagy impairment and lipotoxicity could not be rescued by MCFAs (Fig. 7D to G). These data strongly suggest that increasing MCFA/LCFA ratio protected hepatic cells against LCFA-induced lipotoxicity by rescuing Rubicon-suppressed late-stage autophagy activity.

## Discussion

MCFAs are fatty acids with a carbon chain length of 6–12 carbon atoms and are commonly found in mammalian milk and some plant products such as coconut oil and palm kernel oil. Due to the shorter carbon chain length, MCFAs are difficult to be integrated into triglycerides during chylomicron incorporation in the intestinal tract. Thus, most of the absorbed MCFAs are transported in circulation as free fatty acids^24^. Additionally, MCFAs can enter cells independent of fatty acid binding proteins or transporter proteins and rapidly translocate into mitochondria without the aid of CPT-1 for their β-oxidization^37^. Owing to these metabolic characteristics, MCFAs were found to protect against NAFLD and insulin resistance in rats and humans^25,38,39^. *In vitro* studies also showed that MCFAs attenuated fat accumulation, reactive oxygen species production, and apoptosis caused by LCFAs in human hepatic cells^40,41^. However, the mechanisms by which MCFAs protected against Type 2 diabetes and NASH were not well studied previously.

Recent studies showed that impaired autophagy is associated with NAFLD development, and induction of autophagy can decrease hepatic steatosis in mice^9,16,34,42^. To investigate whether increasing dietary MCFA/LCFA ratio can mitigate HFD-induced Type 2 diabetes and NASH by regulating autophagy, 50% of the lard in mouse HFD was replaced with coconut oil (MCFAD) and fed to mice for 16 weeks. The MCFAD contains approximately same amount of long-chain saturated fatty acids (LCSFAs) compared to SDHFD but halved long-chain unsaturated fatty acids (LCUFAs) replaced by MCFAs. Consistent with previous studies^25,38,39^, our data showed that increasing dietary MCFA ratio significantly improved HFD-induced insulin resistance in mice, but led to more body weight gain (Fig. 1A). Of note, the increased body weight gain was possibly due to attenuated adipose insulin resistance, as we found MCFAs reactivated insulin-induced Akt phosphorylation in adipose tissues of HFD-fed mice (Fig. 1F). The restoration of insulin downstream signaling may mitigate excessive lipolysis in insulin-resistant mice^43^ since we found that the expanding small adipocytes in SDHFD fed mice were replaced by uniformly large adipocytes in MCFAD-fed mice (Fig. 1E). In addition, our data also showed that increasing dietary MCFA ratio attenuated HFD-induced adipose inflammation as the macrophage infiltration was significantly reduced in adipose tissue of MCFAD-fed mice compared to SDHFD-fed mice (Fig. 1E).

We next checked whether the increased MCFAs also mitigated NAFLD development in mice. As shown in Fig. 2A to D, MCFAD-fed mice showed lower hepatic steatosis and fibrosis compared to those fed SDHFD. Of note, the decreased hepatic TG content in MCFAD-fed mice may not be simply caused by inhibition of TG synthesis since the hepatic free fatty acids levels in MCFAD-fed mice were not further increased compared to SDHFD-fed mice (Figure S8). More interestingly, our data showed that increasing the MCFA ratio not only reduced HFD-induced systemic and adipose insulin resistance but also rescued hepatic insulin sensitivity by downregulating basal Akt phosphorylation and enhancing insulin-stimulated Akt phosphorylation (Fig. 2E,F). Consistent with other studies showing autophagy inhibition, ER stress, and apoptosis during NAFLD^6,7,16^, our data showed that increasing MCFA/LCFA ratio restored hepatic autophagic flux and decreased lipotoxicity (Fig. 2G).

To determine whether MCFAs mediated their protective effects against lipotoxicity in a cell-autonomous manner, we treated HepG2 cells with different combinations of LCFAs and MCFAs for 48 hours. Briefly, we treated cells with an equal amount of PA and OA to mimic LCFAs challenge and replaced half of the OA with either CA or LA to increase MCFA/LCFA ratio. Increasing the MCFA/LCFA ratio reduced fat accumulation, insulin resistance, autophagic flux inhibition, ER stress, and apoptosis in HepG2 cells due to long-term LCFAs exposure (Fig. 3). These data indicated that MCFAs were able to directly exert their protective functions on hepatic cells independently from other peripheral tissues. Since previous studies showed that restoration of autophagy attenuates fat accumulation and lipotoxicity^44,45^, we examined whether MCFAs protect against NASH by regulating hepatic autophagy. Interestingly, although MCFAs did not affect basal autophagy in hepatic cells (data not shown), in the context of autophagy inhibition by chronic administration of LCFAs, MCFAs were able to stimulate autophagy (Fig. 3D,E). Moreover, when autophagic flux in HepG2 cells was blocked by either Bafilomycin A1 or chloroquine, the protective effects of MCFAs were eliminated (Fig. 4).

Although previous studies reported activation of AMPK/mTOR/ULK1-regulated autophagy ameliorated hepatic steatosis^13,46,47^, here we demonstrated that fatty acids could block hepatic autophagic flux independently of regulating AMPK/mTOR/ULK1 signaling during NASH. Both i*n vivo* and *in vitro* data showed that long-term exposure of LCFAs and MCFAs did not alter the expression of phospho-AMPK, phospho-mTOR, and phospho-ULK in mouse livers or HepG2 cells (Fig. 5C,D). It is possible that the AMPK/mTOR/ULK1 signaling is differentially regulated at various NAFLD stages since a recent study reported that inhibition of mTOR signaling during early NAFLD was gradually restored in later stages^16^. It is also worth noting that although short-chain fatty acids (fatty acids with less than 5 carbon chain length) were previously reported to activate mTOR/AMPK-mediated autophagy and regulate cellular lipid metabolism^48–50^, we found that MCFAs restored autophagy in fat-loaded hepatic cells independently of regulating AMPK and mTOR signaling (Fig. 5C,D).

The importance of Rubicon in impairment of autophagy during NAFLD was recently demonstrated^16^, so we next examined whether it was involved in MCFAs-mediated restoration of hepatic autophagy. Both i*n vivo* and *in vitro* data show that the upregulation of Rubicon by LCFAs was reversed by increasing MCFA/LCFA ratio (Fig. 5C,D). Overexpression and knockdown experiments showed that Rubicon inhibited autophagic flux by suppressing late-stage degradation rather than early-stage AMPK/mTOR/ULK1-mediated autophagy induction in HepG2 cells (Fig. 6). Rubicon knockdown also protected HepG2 cells from autophagy impairment, ER stress and apoptosis induced by LCFAs (Fig. 7A,B). Interestingly, we found that Rubicon KD also increased *SQSTM1/p62* mRNA expression in fat-loaded HepG2 cells (Fig. 7C), which could be beneficial for maintaining autophagic function during prolonged stressful conditions^51^. More importantly, we demonstrated that increasing MCFA/LCFA ratio did not protect Rubicon-overexpressed HepG2 cells against lipid accumulation, insulin resistance, autophagy impairment, and lipotoxicity caused by LCFAs (Fig. 7D to G). Collectively these data indicated that down-regulation of Rubicon was a prerequisite for MCFAs to rescue autophagy in fat-loaded hepatic cells and could be a potential target for drug therapy for NAFLD.

In summary, we have shown that increasing dietary MCFA/LCFA ratio mitigates HFD-induced Type 2 diabetes and NASH by restoring Rubicon-suppressed late-stage autophagy in the liver. Our study also highlights the importance of carbon chain length in addition to saturation in fatty acid-induced lipotoxicity during the development of Type 2 diabetes and NAFLD. This study provides new insights into the pathogenesis of NAFLD and potential preventive and therapeutic strategies for this condition.

## Methods

### Chemicals and antibodies

Insulin, glucose, Nile Red, Hoechst 33342, BSA, capric acid, lauric acid, palmitic acid, and oleic acid, were purchased from Sigma-Aldrich. Bafilomycin A1 was purchased from Tocris. Antibodies purchased from Cell Signaling Technology, Abcam, and Santa Cruz Biotechnology were listed in Supplementary Table 1.

### HFD-induced Type 2 diabetes and NASH in mice

8-week-old male C57BL/6 mice obtained from the Laboratory Animal Center, College of Medicine, National Taiwan University were fed with either low-fat control diet (CTD), standard high-fat diet (SDHFD), or MCFA-rich high-fat diet (MCFAD) for 16 weeks. The CTD (D12450J, 10% kcal from fat), SDHFD (D12492, 60% kcal from fat), and customized MCFAD were purchased from Research Diets. The MCFAD was made by replacing half the lard in the D12492 diet with hydrogenated coconut oil. Briefly, the MCFAD contains approximately same amount of long-chain saturated fatty acids (LCSFAs) compared to SDHFD but halved long-chain unsaturated fatty acids (LCUFAs), which is replaced by MCFAs. Detailed formulas of diets used in this study are shown in Table 1. All the use and operations on animals were followed by National Institutes of Health guide for the care and use of Laboratory animals (NIH Publications No. 8023, revised 1978), and approved by the National Taiwan University Institutional Animal Care and Use Committee. Animals were maintained at 25 ± 2 °C, approximately 50–60% relative humidity, with a 12–12 hours light-dark cycle and freely accessed water and feed.

**Table 1:** Formulas of diets used in animal studies.

Product No.	**CTD**		**SDHFD**		**MCFAD**	
	D12450J		D12492		N/A	
	**% kcal**					
Protein	20		20		20	
Carbohydrate	70		20		20	
Fat	10		60		60	
Total	100		100		100	
kcal/gm	3.85		5.24		5.24	
**Ingredient**	**gm**	**kcal**	**gm**	**kcal**	**gm**	**kcal**
Casein	200	800	200	800	200	800
L-Cystine	3	12	3	12	3	12
Corn Starch	506.2	2025	0	0	0	0
Maltodextrin 10	125	500	125	500	125	500
Sucrose	68.8	275	68.8	275	68.8	275
Cellulose, BW200	50	0	50	0	50	0
Soybean Oil	25	225	25	225	25	225
Lard	20	180	245	2205	106	956
Hydrogenated Coconut Oil	0	0	0	0	138.8	1249

### Oral glucose tolerance test

6-hour starved mice were orally administrated a single dose of glucose (2 g/kg body weight). Before and at the 15, 30, 45, 60, 90, and 120 minute time points after glucose gavage, blood from mouse tails were collected and analyzed using Bayer’s Elite Glucometer with test strips. Blood glucose data were then plotted and calculated as area under curve values for statistical analyses.

### Histological section staining and adipocyte size quantification

Mouse liver and adipose tissues were fixed, embedded in paraffin, and trimmed as 5-μm-thick sections. After the sections were made, H&E and Sirius Red staining were performed following standard protocols. To quantify the adipocyte size, images of H&E-stained adipose sections were randomly taken from each section and then analyzed using Adiposoft software^52^. Default parameters in Adiposoft were set for auto-detection of adipocytes.

### Liver triglyceride assay

For hepatic triglyceride (TG) measurement, assay Kit (K622) from BioVision was used following the manufacturer’s protocol.

### Western blotting analyses

Mouse liver tissues were homogenized and extracted in Pierce IP Lysis Buffer (87787) added with Complete EDTA-free Protease Inhibitor Cocktail and PhosSTOP (Roche) for protein quantification. After protein estimation, samples were diluted and mixed with Laemmli Sample Buffer (Bio-Rad) and. For *in vitro* samples, cells were directly lysed and harvested in Laemmli buffer. Protein lysates were boiled at 98 °C for 5 minutes and subjected to Western blotting analysis as previously described^53^.

### Dot blotting for liver collagen I quantification

For Dot blotting analysis, undenatured liver lysates were carefully dotted on activated methanol membranes (20 μg proteins per dot) and fixed by air-drying for 1.5 hours at RT. Dotted membranes were then blocked, incubated with anti-collagen I antibody and secondary HRP-conjugated antibody, and visualized as in Western blotting analysis.

### Liver Hydroxyproline Content Assay

To determine the liver hydroxyproline levels, commercial assay kit purchased from BioVision (K226) were used following manufacture recommended protocol.

### *In vitro* cell culture

HepG2 cells were purchased from Taiwan Bioresource Collection and Research Center (BCRC number: RM60025) and cultured in DMEM medium (Sigma-Aldrich) supplemented with 10% FBS and 1% penicillin-streptomycin (Gibco). Cells were maintained at 37 °C with 5% CO_2_. To make the fatty-acid-containing medium, fatty acid stocks (100 mM in ethanol) were added into 1% BSA-containing medium and incubated for 30 minutes at 37 °C before use. For autophagic flux analyses, cells were treated with or without Bafilomycin A1 (50 nM), for another 3 hours before harvest. Autophagy flux was then quantified by measuring the amount of Bafilomycin A1-induced LC3 II protein accumulation.

To knock down Rubicon, predesigned siGENOME non-targeting siRNA or SMARTpool Rubicon specific siRNAs (Dharmacon) were reversely transfected into HepG2 cells using Lipofectamine RNAiMAX Transfection Reagent (Thermo Scientific). For Rubicon overexpression, the EGFP-Rubicon plasmid (a gift from Qing Zhong, Addgene plasmid # 28022) was reversely transfected into HepG2 cells using Lipofectamine 3000 Reagent (Thermo Scientific). Transfection procedures were carried out following manufacture’s protocol.

### Nile Red staining

To quantify intracellular fat accumulation, fluorescent Nile Red staining was used. Briefly, cells grown in black 96-well plates were fixed with 4% paraformaldehyde and stained with Nile red (1 μg/mL) and Hoechst 33342 (2 μg/mL) in PBS for 30 minutes in the dark. The fluorescence of Nile Red (excitation at 485 nm, emission at 535 nm; when EGFP-Rubicon were overexpressed, excitation at 590 nm and emission at 590 nm were used) and Hoechst 33342 (excitation at 350 nm; emission at 461 nm) was measured using Biotek Synergy H1 Multi-Mode Reader for intracellular fat quantification and cell number normalization respectively.

### RNA extraction and qPCR analyses

Total RNA from mouse livers or HepG2 cells was extracted using TRIzol Reagent (Thermo Scientific) following the manufacturer’s protocol. After RNA extraction, the PrimeScript RT Reagent Kit purchased from Takara Bio was used for first-strand cDNA synthesis. To quantify mRNA expression levels, qPCR analyses were performed using the QuantStudio 3 System with Fast SYBR Green Master Mix reagent (Applied Biosystems) and gene-specific primer pairs (Supplementary Table 2). *ACTB* or *Actb* were used as internal controls.

### Statistical analysis

In this study, statistical analyses were conducted and plots were generated using SigmaPlot software (Version 12.0, Systat Software). *In vivo* (n = 4–6) or *in vitro* data obtained from at least three independent experiments were pooled and expressed as the mean ± standard error of the mean (SEM). Statistically significant differences (*p* < 0.05) were determined by Student’s t-tests or ANOVA followed by Holm-Sidak test.

### Data availability

No datasets were generated or analyzed during the current study.

## Electronic supplementary material

supplementary information
