# Supplementary material for: Increasing Dietary Medium-Chain Fatty Acid Ratio Mitigates High-fat Diet-Induced Non-Alcoholic Steatohepatitis by Regulating Autophagy
Source: Sci Rep. 2017 Oct 25;7:13999. doi: 10.1038/s41598-017-14376-y (PMC5656678; doi:10.1038/s41598-017-14376-y)
Supplement: Supplementary file 1 — supplementary information [file 41598_2017_14376_MOESM1_ESM.pdf]

Increasing Dietary Medium-Chain Fatty Acid Ratio Mitigates High-fat Diet-Induced Non-Alcoholic Steatohepatitis by Regulating Autophagy

Mu-En Wang[1, 2], Brijesh K. Singh[2], Meng-Chieh Hsu[1], Chien Huang[1], Paul M. Yen[2], Leang-Shin Wu[1], De-Shien Jong[1], and Chih-Hsien Chiu[1, *]

[1]Laboratory of Animal Physiology, Department of Animal Science and Technology, National Taiwan University, Taipei 10617, Taiwan.
[2]Laboratory of Hormonal Regulation, Cardiovascular and Metabolic Disorders Program, Duke-National University of Singapore Graduate Medical School, Singapore 16987, Singapore.

[*]**Corresponding Author: Chih-Hsien Chiu**
Address: 50, Lane 155, Sec 3, Keelung Rd., 106 Taipei, Taiwan
Tel.: +886-2-3366-4171; Fax: +886-2-3366-4070 chiuchihhsien@ntu.edu.tw

Supplementary Tables

Table 1. Antibodies used in Western Blotting and Dot Blotting

Antibody Name	Company	Product Number
Anti-LC3	Cell Signaling Technology	#2775
Anti-phospho-Akt Ser473	Cell Signaling Technology	#4060
Anti-Akt	Cell Signaling Technology	#4691
Anti-CHOP (for in vivo samples)	Cell Signaling Technology	#5554
Anti-CHOP	Cell Signaling Technology	#2895
Anti-cleaved caspase-3	Cell Signaling Technology	#9661
Anti-phospho-mTOR Ser2448	Cell Signaling Technology	#5536
Anti-mTOR	Cell Signaling Technology	#2983
Anti-phospho-p70S6K Thr389	Cell Signaling Technology	#9234
Anti-p70S6K	Cell Signaling Technology	#2708
Anti-phospho-ULK1 Ser757	Cell Signaling Technology	#14202
Anti-phospho-ULK1 Ser555	Cell Signaling Technology	#5869
Anti-ULK1	Cell Signaling Technology	#8054
Anti-phospho-AMPK Thr172	Cell Signaling Technology	#2535
Anti-AMPK	Cell Signaling Technology	#2532
Anti-Beclin-1	Cell Signaling Technology	#3495
Anti-ATG5	Cell Signaling Technology	#12994
Anti-Rubicon	Cell Signaling Technology	#8465
Anti-β-actin (for *in vivo* samples)	Cell Signaling Technology	#8457
Anti-Collagen I	abcam	ab34710
Anti-SQSTM1/p62	abcam	ab109012
Anti-phospho-GSK-3β Ser9	Cell Signaling Technology	#5558
Anti-GSK-3β	Cell Signaling Technology	#3915
Anti-β-actin	Santa Cruz Biotechnology	sc-47778
Goat anti-rabbit IgG-HRP	Santa Cruz Biotechnology	sc-2004
Goat anti-mouse IgG-HRP	Santa Cruz Biotechnology	sc-2005

Table 2. Primers used in qPCR analyses

Gene name	Forward (5’ to 3’)	Reverse (5’ to 3’)
Map1lc3b	GGAGCTTTGAACAAAGAGTGGAA	GGTCAGGCACCAGGAACTTG
Sqstm1/p62	CCTTGCCCTACAGCTGAGTC	CCTCAATGCCTAGAGGGCTG
Ulk1	AAACATCGTGGCGCTGTATG	CGCATAGTGTGCAGGTAGTCA
Becn1	AGGAACTCACAGCTCCATTACT	ACCATCCTGGCGAGTTTCAAT
Actb	CACTGTCGAGTCGCGTCCA	CATCCATGGCGAACTGGTGG
MAP1LC3B	CCGCACCTTCGAACAAAGAG	AAGCTGCTTCTCACCCTTGT
SQSTM1/p62	AGAATCAGCTTCTGGTCCATCG	TTCTTTTCCCTCCGTGCTCC
ULK1	AGAACCTCGCCAAGTCTCAG	ACCGTTGCAGTACTCCATAACC
BECN1	CCAGGAACTCACAGCTCCATT	TCTGCGAGAGACACCATCCT
RUBCN	TCTCATGCAAACTGATGAAC	AGTTTGTGAAAGACATTCGG
ACTB	GAGCACAGAGCCTCGCCTTT	TCATCATCCATGGTGAGCTGG

Supplementary Figure Legends

Figure S1. The full-length blot of p-Akt, Akt presented in Figure 1 of the main text.

Figure S2. The full-length blot of collagen I, p-Akt, Akt, SQSTM1/p62, LC3, CHOP, cleaved caspase 3, and β-actin presented in Figure 2 of the main text.

Figure S3. The full-length blot of p-Akt, Akt, SQSTM1/p62, LC3, CHOP, cleaved caspase 3, and β-actin presented in Figure 3 of the main text.

Figure S4. The full-length blot of p-Akt, Akt, SQSTM1/p62, LC3, CHOP, cleaved caspase 3, and β-actin presented in Figure 4 of the main text.

Figure S5. The full-length blot of p-AMPK, AMPK, p-mTOR, mTOR, p-p70S6K, p70S6K, p-ULK1, ULK1, Beclin-1, ATG5, Rubicon, and β-actin presented in Figure 5 of the main text.

Figure S6. The full-length blot of EGFP-Rubicon, Rubicon, p-AMPK, AMPK, p-mTOR, mTOR, p-p70S6K, p70S6K, p-ULK1, ULK1, Beclin-1, ATG5, SQSTM1/p62, LC3, and β-actin presented in Figure 6 of the main text.

Figure S7. The full-length blot of Rubicon, SQSTM1/p62, LC3, CHOP, cleaved caspase 3, EGFP-Rubicon, p-Akt, Akt, and β-actin presented in Figure 7 of the main text.

Figure S8. The free fatty acid levels in mice fed with either CTD, SDHFD, or MCFAD for 16 weeks. Values are mean ± SEM. (n=6). * indicates statistical significance, $P < 0.05$. n.s.: no significant difference.

Figure S9. The Representative immunoblots and densitometric quantification of phospho-GSK-3β (Ser9) in fat-loaded HepG2 cells treated with or without insulin **(A)**, and the calculated insulin-stimulated GSK-3β phosphorylation fold change results **(B).** Values are mean ± SEM (n=3). *, # (vs. BSA treated cells without insulin stimulation), and † (vs. SDF-treated cells without insulin stimulation) indicate statistical significance, $P < 0.05$. n.s.: no significant difference.

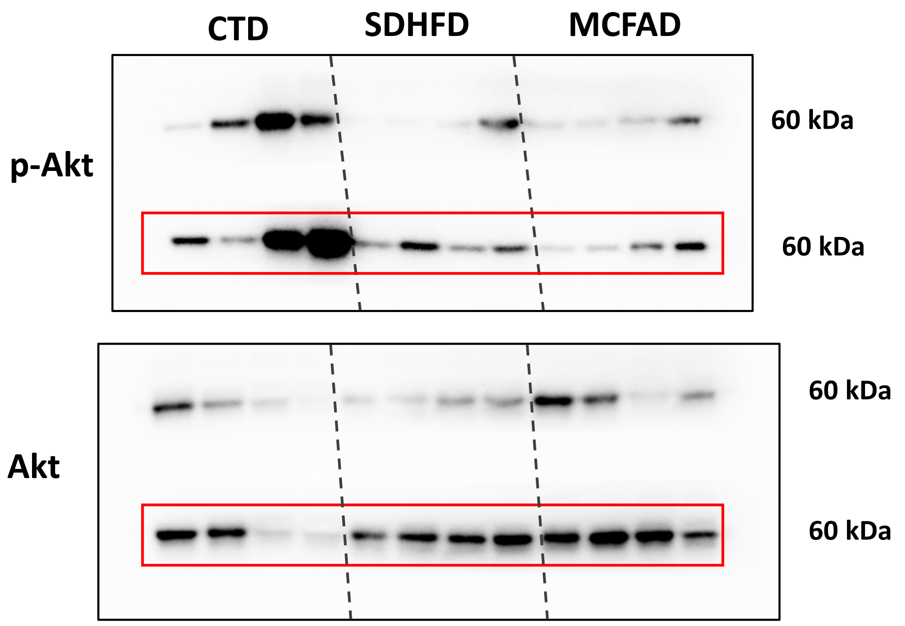

Figure S1

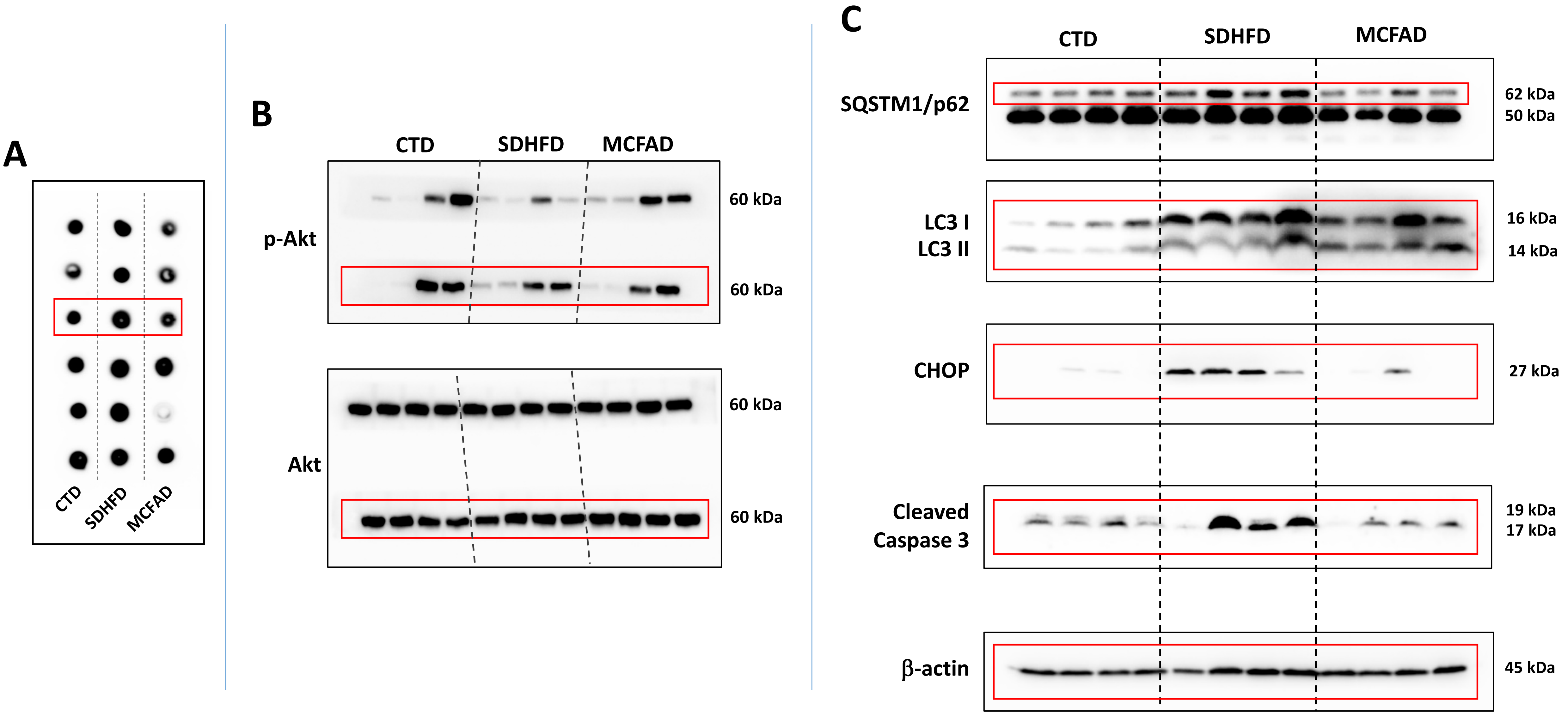
A
CTD
SDHFD
MCFAD
B
CTD
SDHFD
MCFAD
p-Akt
60 kDa
60 kDa
Akt
60 kDa
60 kDa
C
CTD
SDHFD
MCFAD
SQSTM1/p62
62 kDa
50 kDa
LC3 I
LC3 II
16 kDa
14 kDa
CHOP
27 kDa
Cleaved
Caspase 3
19 kDa
17 kDa
β-actin
45 kDa

Figure S2

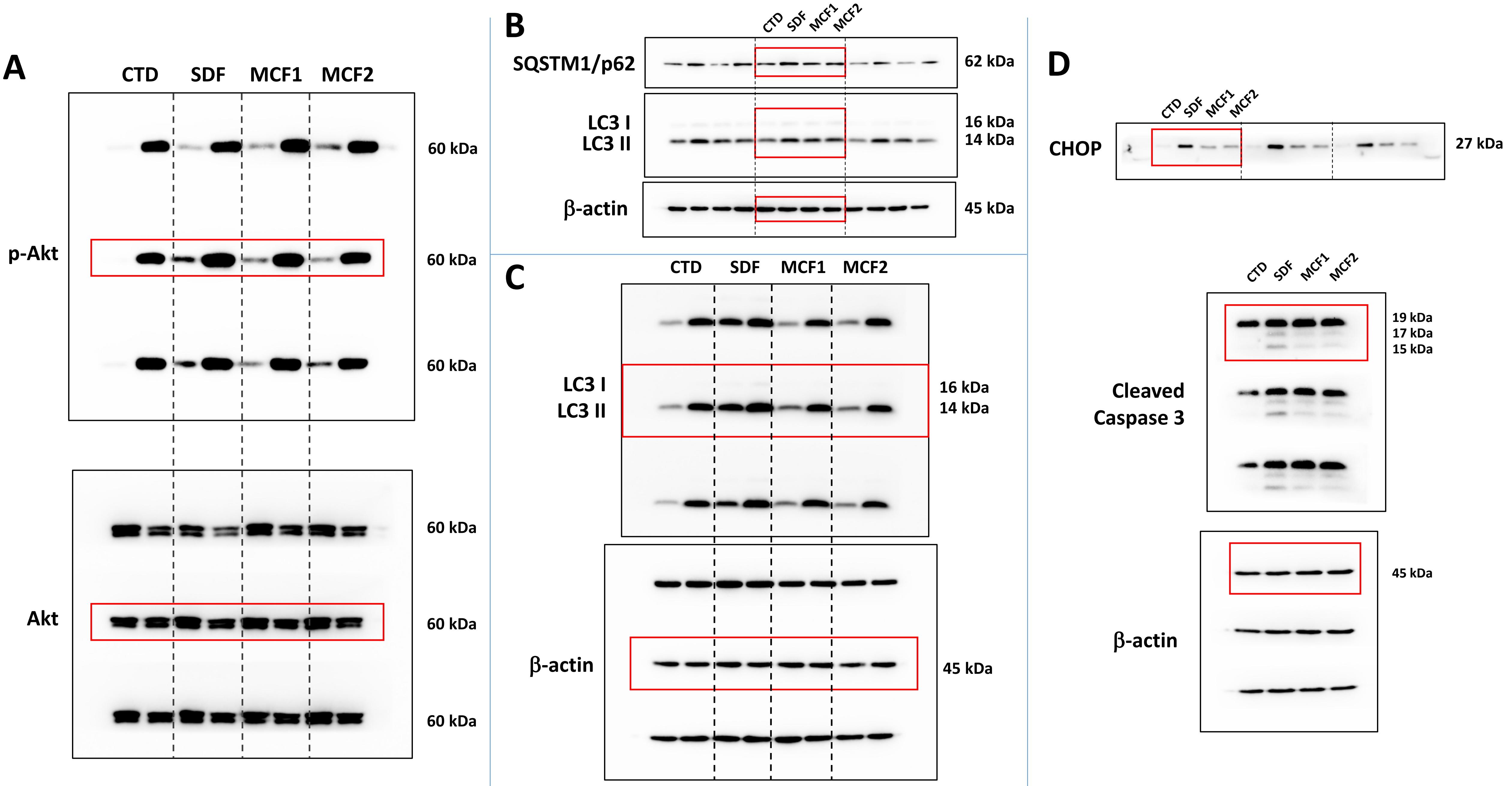
A
CTD
SDF
MCF1
MCF2
p-Akt
60 kDa
60 kDa
60 kDa
Akt
60 kDa
60 kDa
60 kDa
B
CTD
SDF
MCF1
MCF2
SQSTM1/p62
62 kDa
LC3 I
LC3 II
16 kDa
14 kDa
β-actin
45 kDa
C
CTD
SDF
MCF1
MCF2
LC3 I
LC3 II
16 kDa
14 kDa
β-actin
45 kDa
D
CTD
SDF
MCF1
MCF2
CHOP
27 kDa
CTD
SDF
MCF1
MCF2
Cleaved
Caspase 3
19 kDa
17 kDa
15 kDa
β-actin
45 kDa

Figure S3

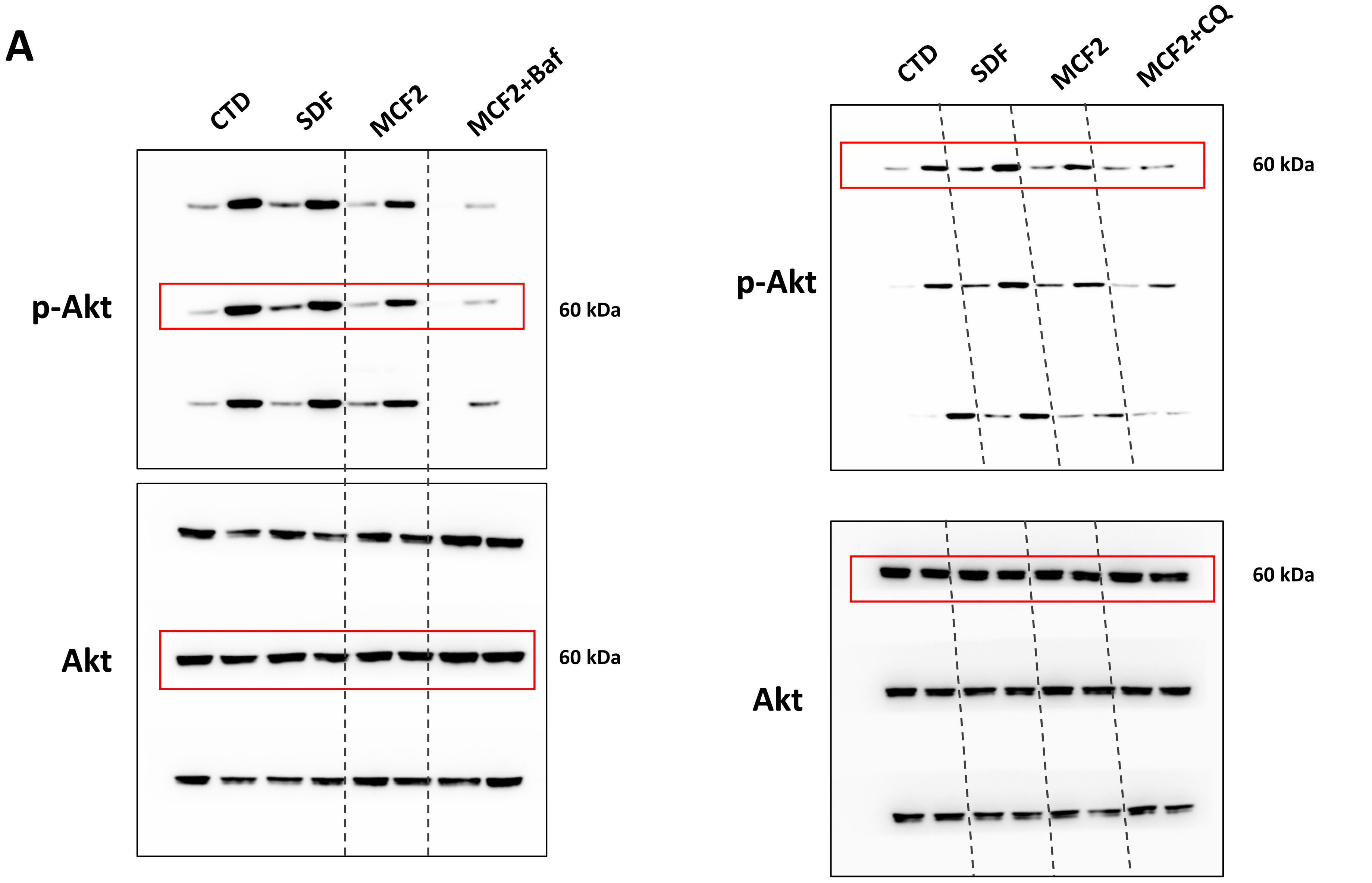
A
CTD
SDF
MCF2
MCF2+Baf
p-Akt
60 kDa
Akt
60 kDa
CTD
SDF
MCF2
MCF2+CQ
p-Akt
60 kDa
Akt
60 kDa

Figure S4

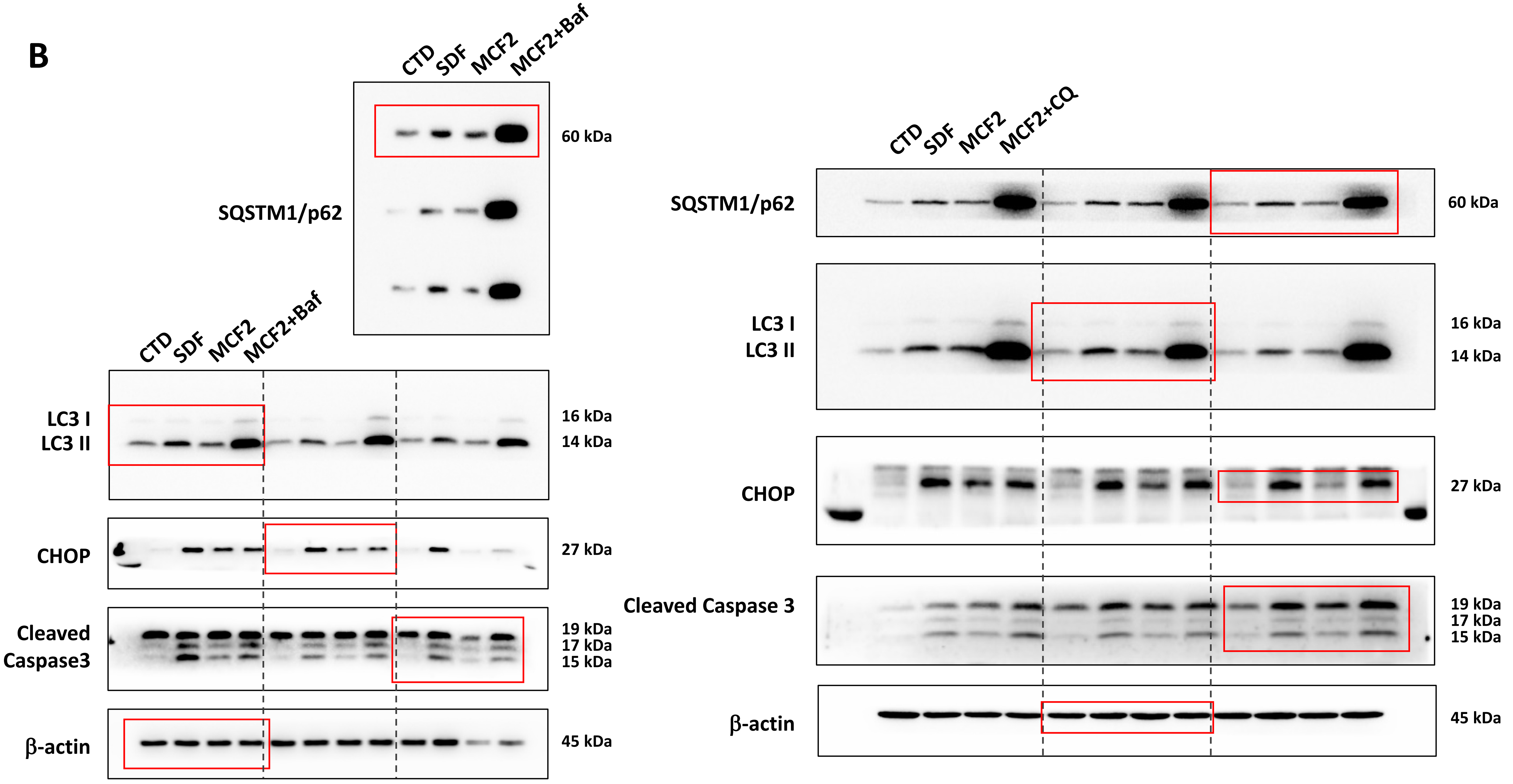

Figure S4 (continued)

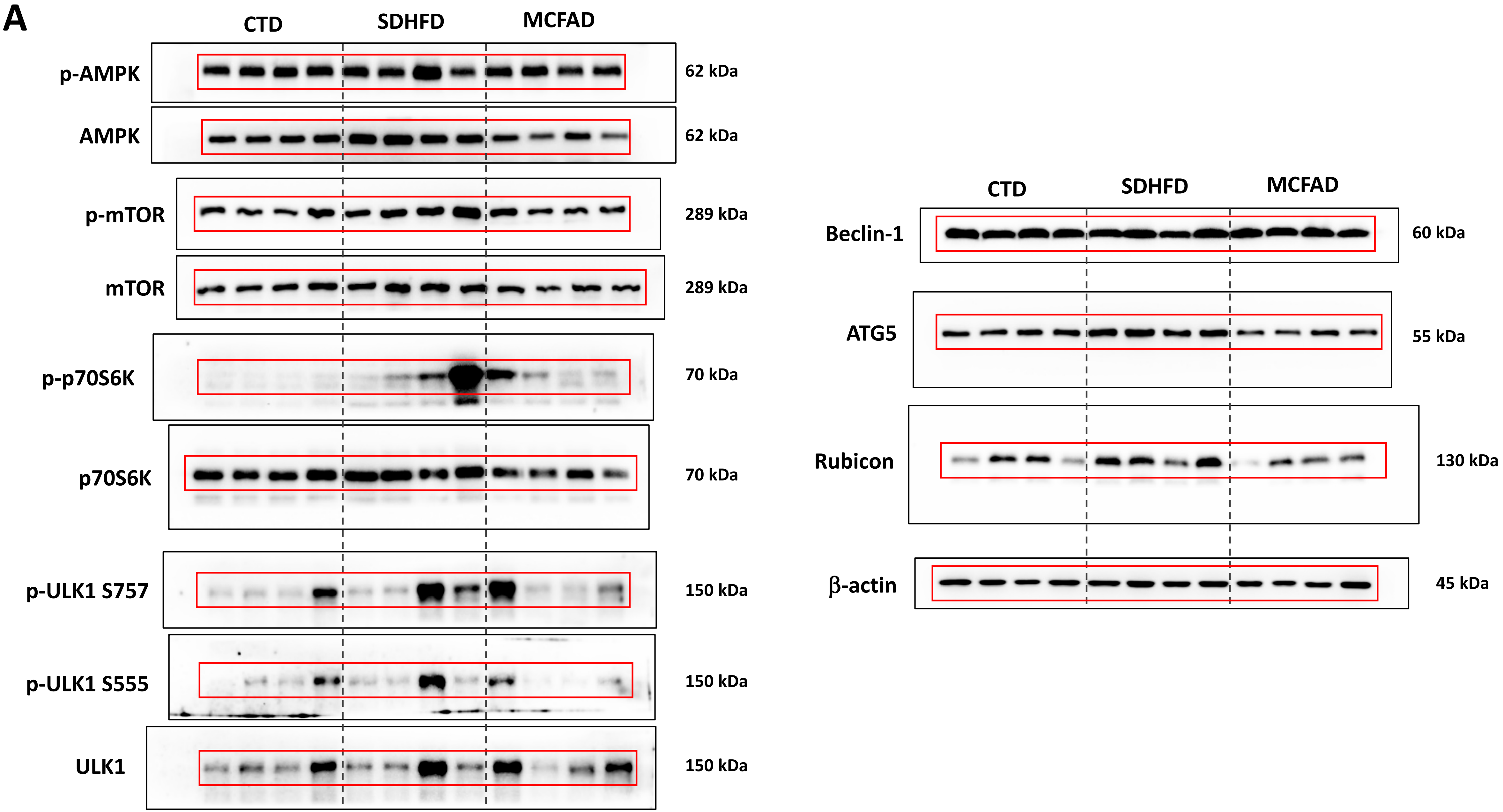
A
CTD
SDHFD
MCFAD
p-AMPK
62 kDa
AMPK
62 kDa
p-mTOR
289 kDa
mTOR
289 kDa
p-p70S6K
70 kDa
p70S6K
70 kDa
p-ULK1 S757
150 kDa
p-ULK1 S555
150 kDa
ULK1
150 kDa
CTD
SDHFD
MCFAD
Beclin-1
60 kDa
ATG5
55 kDa
Rubicon
130 kDa
β-actin
45 kDa

Figure S5

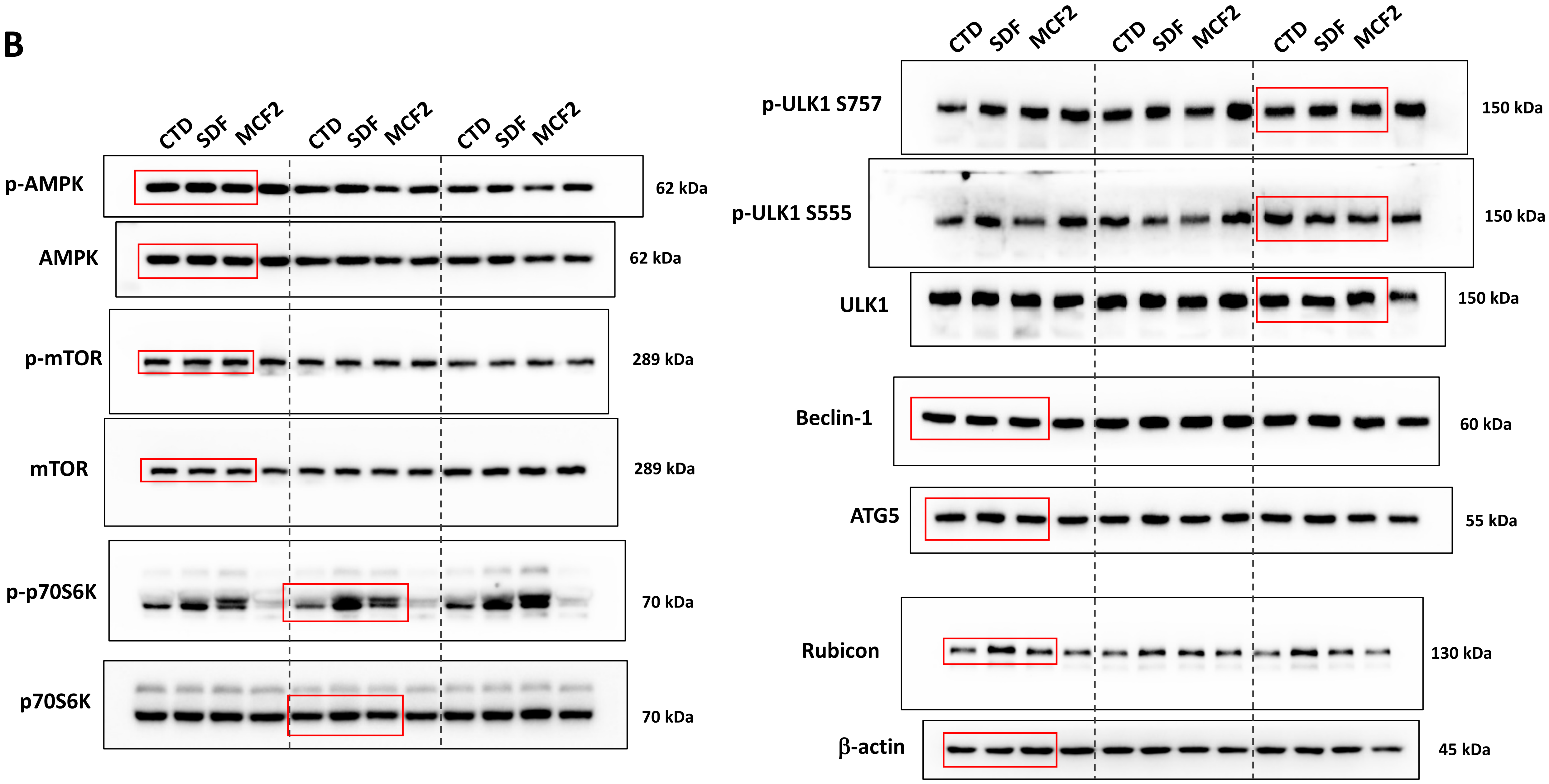

Figure S5 (Continued)

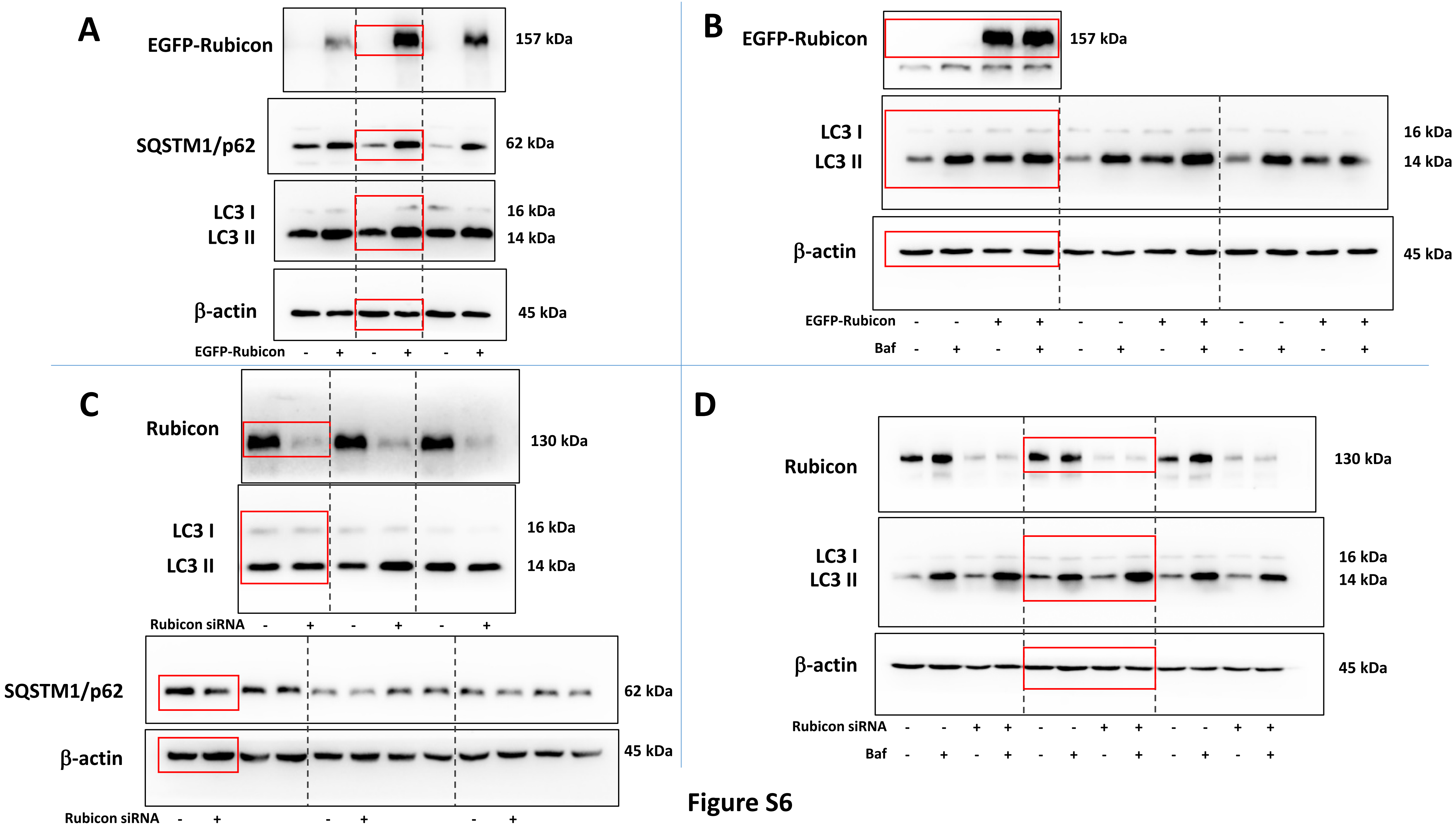
A
EGFP-Rubicon
157 kDa
SQSTM1/p62
62 kDa
LC3 I
LC3 II
16 kDa
14 kDa
β-actin
45 kDa
EGFP-Rubicon - + - + - +
B
EGFP-Rubicon
157 kDa
LC3 I
LC3 II
16 kDa
14 kDa
β-actin
45 kDa
EGFP-Rubicon - - + + - - + + - - + +
Baf - + - + - + - + - + - +
C
Rubicon
130 kDa
LC3 I
LC3 II
16 kDa
14 kDa
Rubicon siRNA - + - + - +
SQSTM1/p62
62 kDa
β-actin
45 kDa
Rubicon siRNA - + - + - +
D
Rubicon
130 kDa
LC3 I
LC3 II
16 kDa
14 kDa
β-actin
45 kDa
Rubicon siRNA - - + + - - + + - - + +
Baf - + - + - + - + - + - +

Figure S6

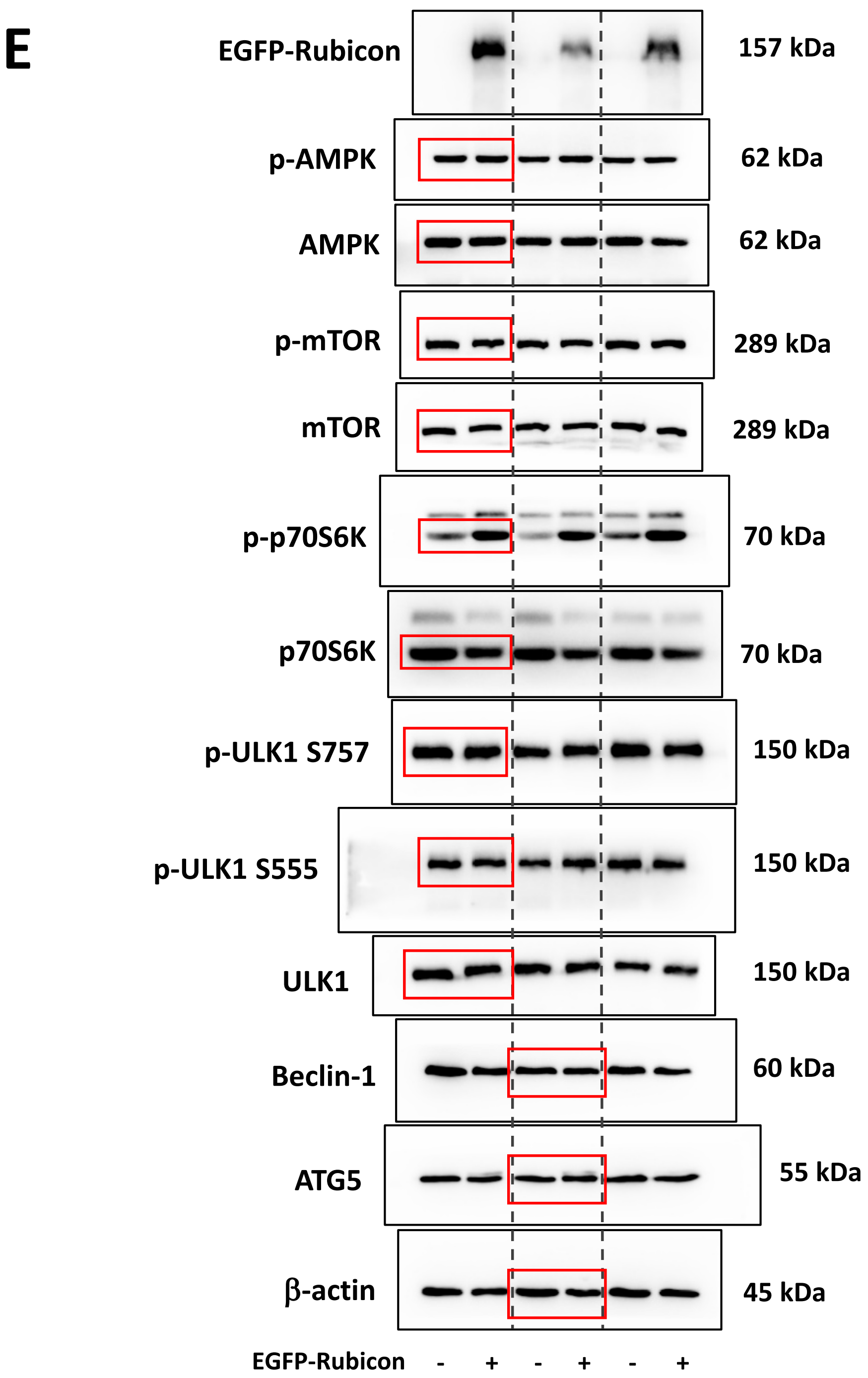

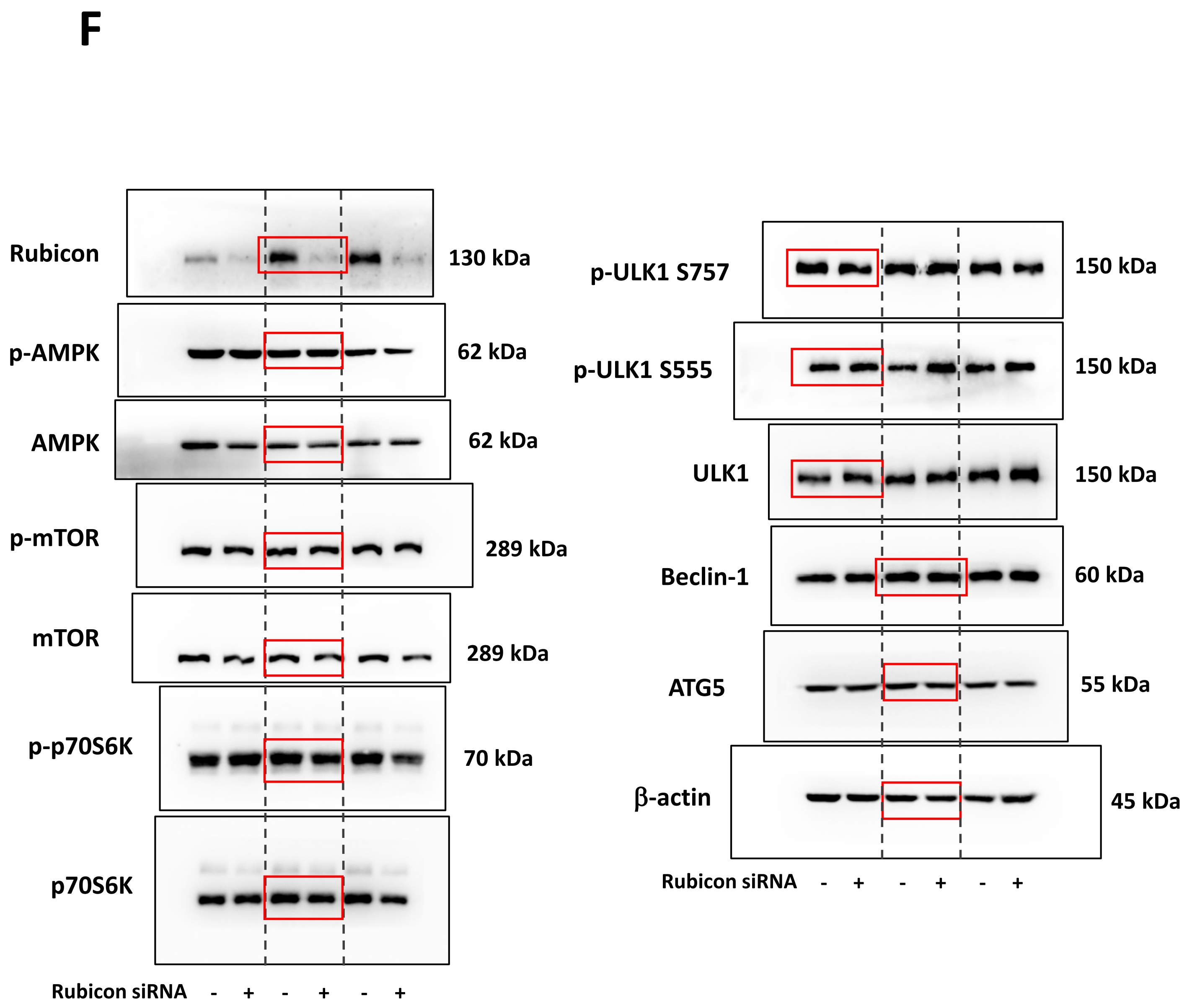

Figure S6 (Continued)

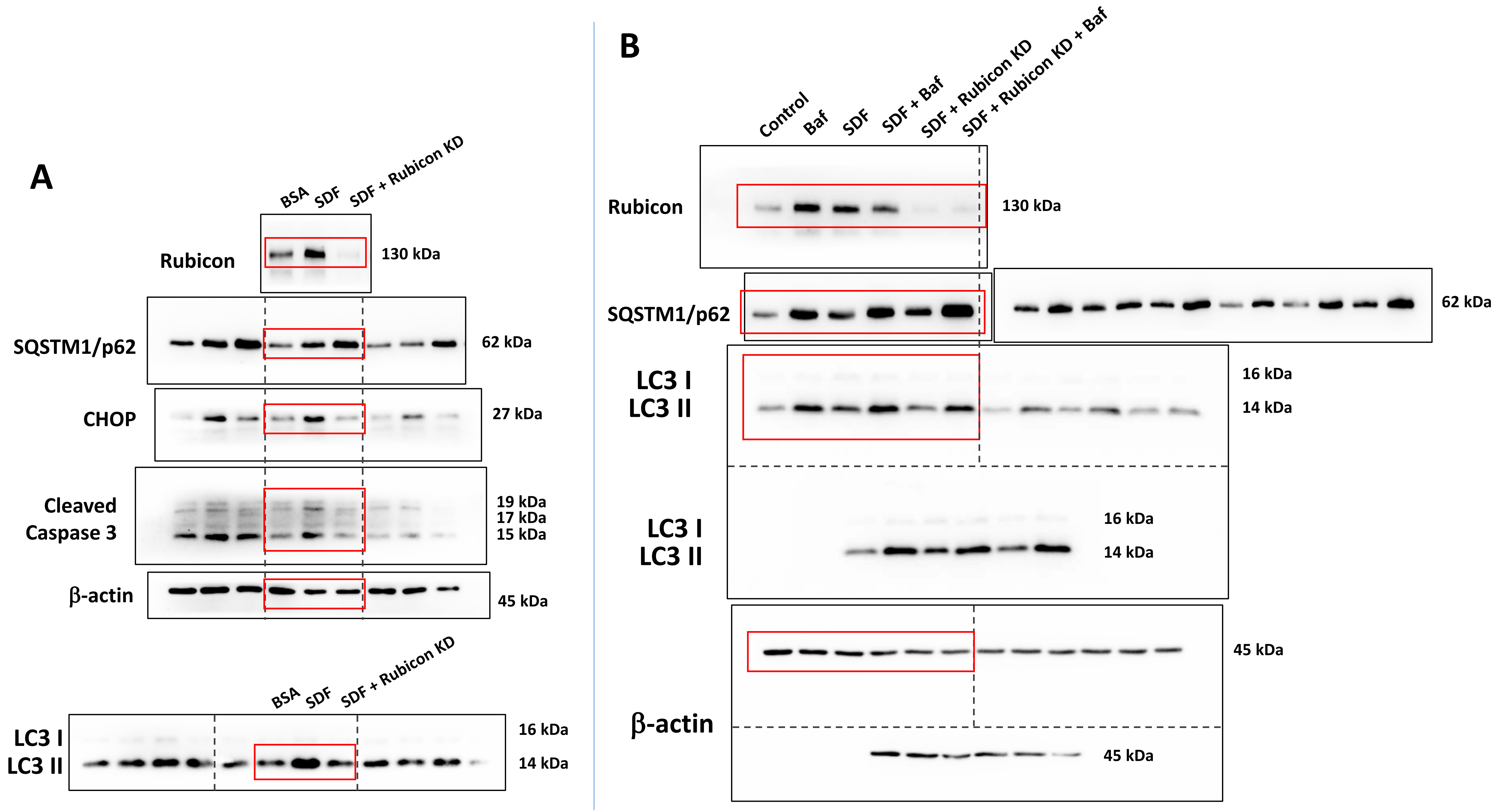
A
BSA
SDF
SDF + Rubicon KD
Rubicon
130 kDa
SQSTM1/p62
62 kDa
CHOP
27 kDa
Cleaved Caspase 3
19 kDa
17 kDa
15 kDa
β-actin
45 kDa
LC3 I
LC3 II
16 kDa
14 kDa
B
Control
Baf
SDF
SDF + Baf
SDF + Rubicon KD
SDF + Rubicon KD + Baf
Rubicon
130 kDa
SQSTM1/p62
62 kDa
LC3 I
LC3 II
16 kDa
14 kDa
β-actin
45 kDa

Figure S7

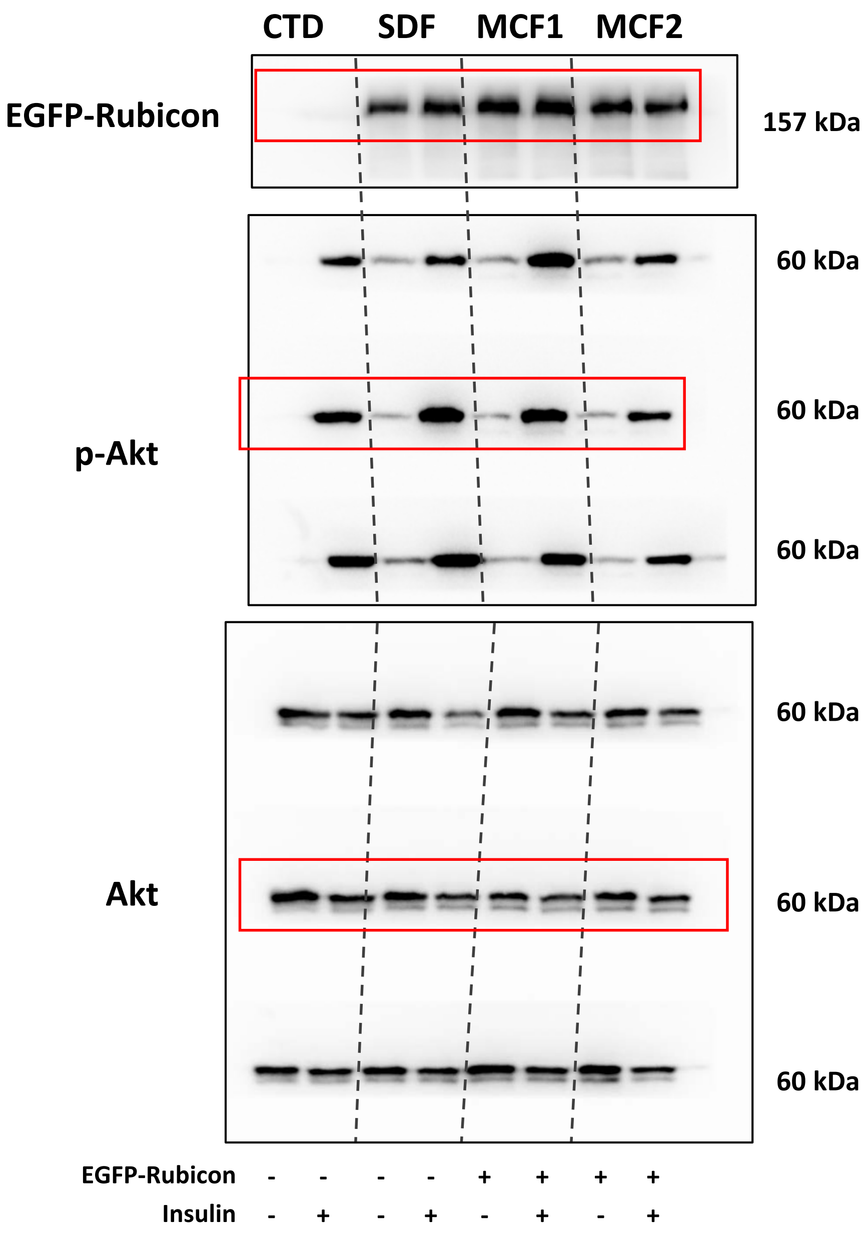

Figure S7 (Continued)

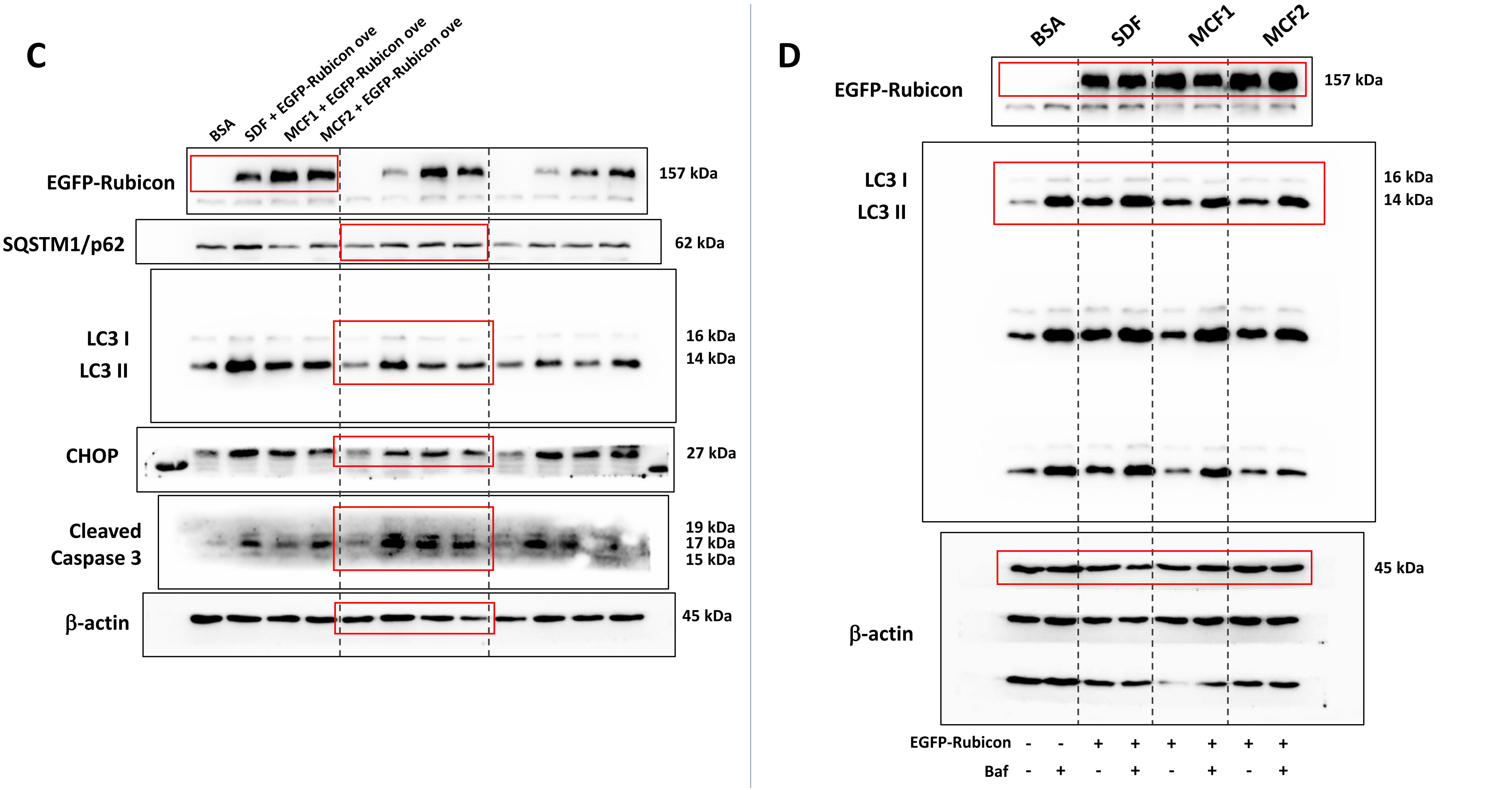

Figure S7 (Continued)

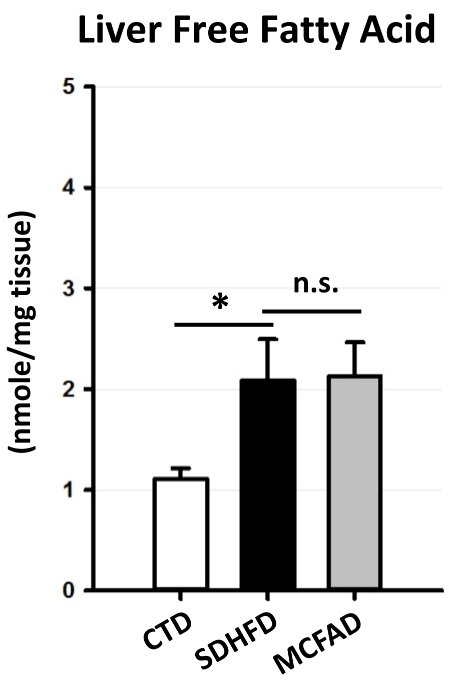
Liver Free Fatty Acid
(nmole/mg tissue)
5
4
3
2
1
0
*
n.s.
CTD
SDHFD
MCFAD

Figure S8

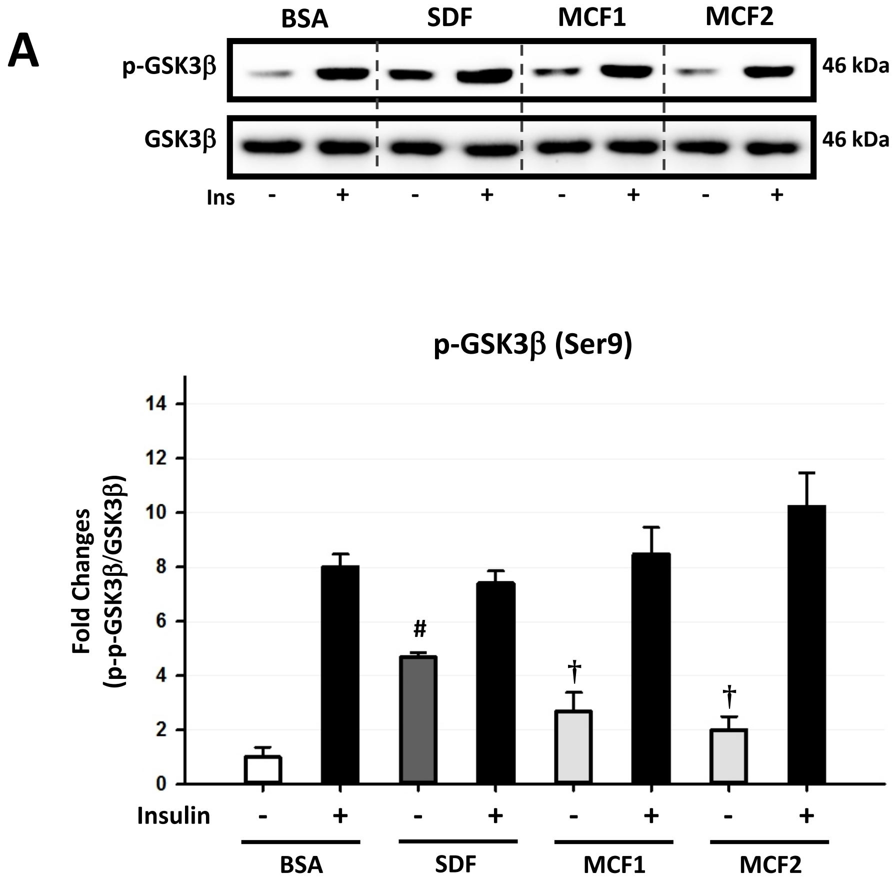
A
BSA
SDF
MCF1
MCF2
p-GSK3β
46 kDa
GSK3β
46 kDa
Ins
- + - + - + - +
p-GSK3β (Ser9)
Fold Changes
(p-p-GSK3β/GSK3β)
14
12
10
8
6
4
2
0
#
†
†
Insulin
- + - + - + - +
BSA
SDF
MCF1
MCF2

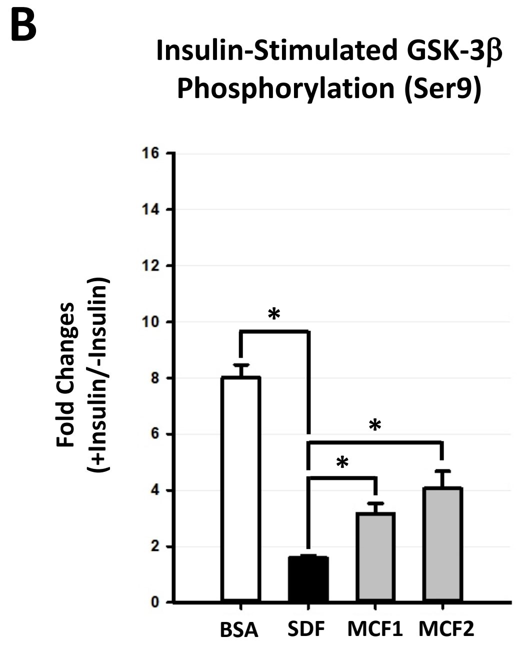
B
Insulin-Stimulated GSK-3β
Phosphorylation (Ser9)
Fold Changes
(+Insulin/-Insulin)
16
14
12
10
8
6
4
2
0
*
*
*
BSA
SDF
MCF1
MCF2

Figure S9
